# Quantum optical measurements with undetected photons through vacuum field indistinguishability

**DOI:** 10.1038/s41598-017-06800-0

**Published:** 2017-07-26

**Authors:** Sun Kyung Lee, Tai Hyun Yoon, Minhaeng Cho

**Affiliations:** 1 Center for Molecular Spectroscopy and Dynamics, Institute for Basic Science, Seoul, 02841 Republic of Korea; 2 Department of Physics, Korea University, Seoul, 02841 Republic of Korea; 3 Department of Chemistry, Korea University, Seoul, 02841 Republic of Korea

## Abstract

Quantum spectroscopy and imaging with undetected idler photons have been demonstrated by measuring one-photon interference between the corresponding entangled signal fields from two spontaneous parametric down conversion (SPDC) crystals. In this Report, we present a new quantum optical measurement scheme utilizing three SPDC crystals in a cascading arrangement; here, neither the detection of the idler photons which interact with materials of interest nor their conjugate signal photons which do not interact with the sample is required. The coherence of signal beams in a single photon W-type path-entangled state is induced and modulated by indistinguishabilities of the idler beams and crucially the quantum vacuum fields. As a result, the optical properties of materials or objects interacting with the idler beam from the first SPDC crystal can be measured by detecting second-order interference between the signal beams generated by the other two SPDC crystals further down the set-up. This gedankenexperiment illustrates the fundamental importance of vacuum fields in generating an optical tripartite entangled state and thus its crucial role in quantum optical measurements.

## Introduction

Conventionally, spectroscopy measurements are described in a semi-classical manner where the light-matter interaction leading to transitions between quantized atomic or molecular energy levels use classical incident fields to ascertain the transition probabilities^1, 2^. Crucially, fields that interact with the sample must be detected directly at the intensity level so practical limitations may exist for measurements depending on, for example, photon energy due to frequency dependent detector sensitivity. Recently, quantum spectroscopy with quantum entangled photons has been proven to overcome various limits of the classical spectroscopy^3, 4^. In addition, sub-shot-noise quantum imaging is possible even with sub-Rayleigh resolution^5^. Such quantum spectroscopy and imaging are possible through detection of temporal correlation or coincidence counting rate of the entangled pair of signal and idler photons generated by spontaneous parametric down conversion (SPDC)^6, 7^. Since the early investigations^8–10^ on spatial and temporal correlations between signal and idler photons from a single SPDC process, variations on this single SPDC scheme, such as quantum correlations of frequency non-degenerate signal and idler photons or degenerate twin photons, have been used to test the fundamental tenets of quantum mechanics^11^.

## Description of triple-SPDC scheme

**Figure 1:**
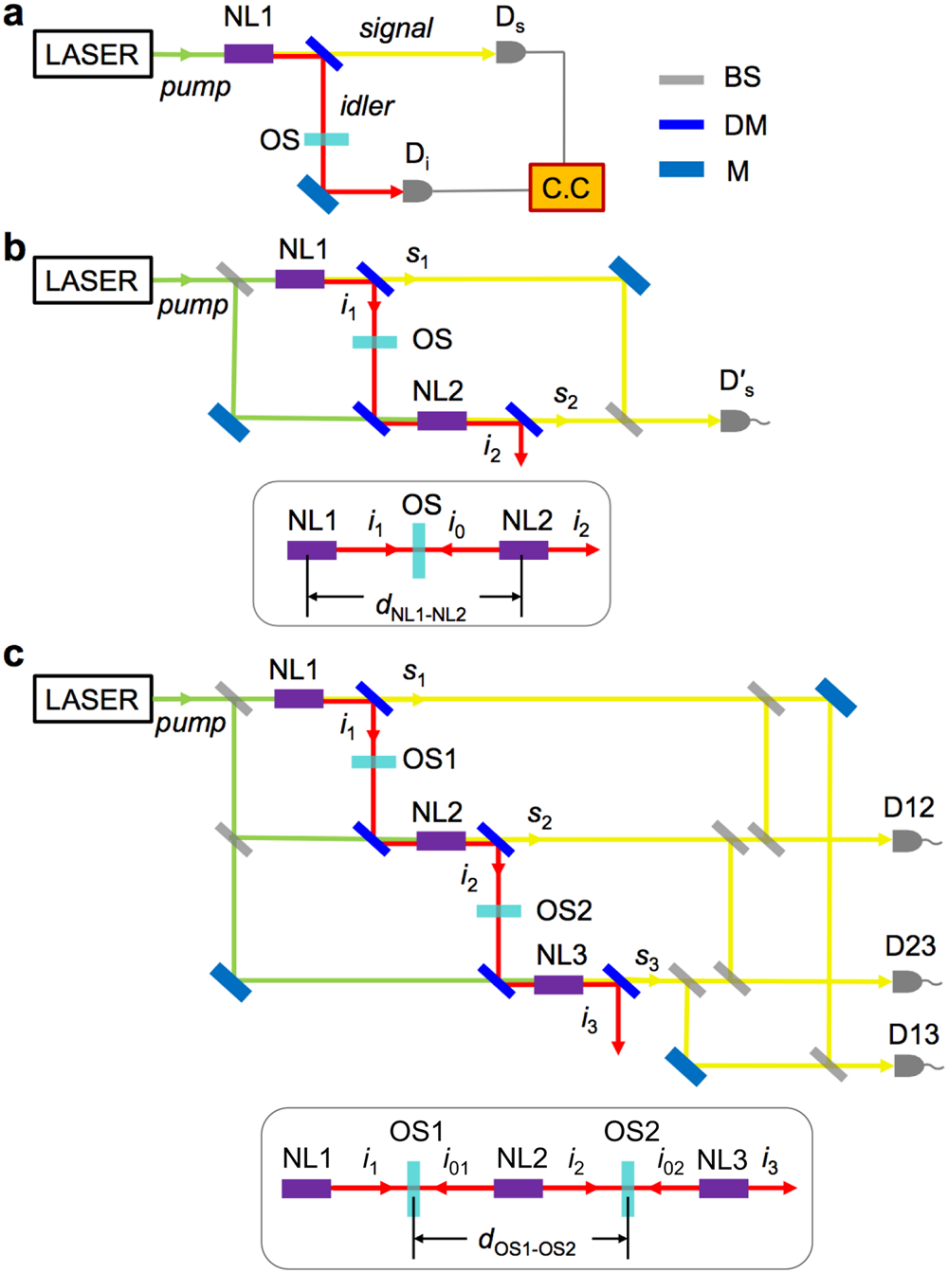
Schematic representations of single-, double-, and triple-SPDC experiments. Pump, signal, and idler beams are shown in green, red, and yellow in color, respectively. NL, OS, D, M, and C.C. represent nonlinear crystal, optical sample, detector, mirror, and coincidence counter, respectively. (**a**) Single-SPDC experimental setup with coincidence counting rate measurements with *D*_*s*_ and *D*_*i*_. (**b**) Double-SPDC experimental setup with two NL crystals. One-photon interference (second-order interference) between *s*_1_ and *s*_2_ is detected by ${D}_{s}^{{\rm{^{\prime} }}}$. (**c**) Triple-SPDC gedankenexperimental setup with three NL crystals. One-photon interference (second-order interference) between *s*_1_ and *s*_2_, between *s*_1_ and *s*_3_, and between *s*_2_ and *s*_3_ are detected by D12, D13, and D23, respectively. The inset figures of (**b** and **c**) represent a one-dimensional representation of idler beam pathway along its propagation axis.

In an experiment using single-SPDC crystal (Fig. 1a), pumping of a nonlinear (NL) crystal lacking an inversion symmetry results in the parametric generation of a low intensity signal and idler photon pair that are in a correlated (quantum entangled) state. A two-photon interference, i.e., fourth-order (in the field) interference, between signal and idler photons, manifested by coincidence counting rate measurements^12^, results from quantum correlation between the two, even though the signal and idler beams are mutually incoherent^13^. If the idler beam interacts with atoms or molecules that absorb idler photons, the measured coincidence counting rate is accordingly attenuated. This is the basis of quantum spectroscopy that requires temporal correlation detections (or two-photon interference measurements) of both signal and idler photons^14, 15^.

Mandel and coworkers further considered an interesting double-SPDC scheme, where two NL crystals are arranged in a cascading geometry (Fig. 1b)^16^. Since the idler beam, *i*_1_, from the first NL crystal, NL1, is aligned to traverse the second NL crystal in a collinear way, the two idler beams *i*_1_ and *i*_2_ are indistinguishable, which in turn induces a quantum coherence between two conjugate *signal* beams^17^. This scheme is closely related to quantum eraser experiment in that it recovers the interference fringe from the signal photons by erasing “which-way” information of the idler photons^18, 19^. However, the need for coincidence detection between signal and idler photons is removed by perfect overlap of the two idler modes^20^. Optical component such as a beam splitter, neutral density filter, phase object, absorbing material or sample, placed between the two NL crystals would attenuate the degree of coherence of the two signal beams (*s*_1_ and *s*_2_) or their second-order interference as the transmissivity amplitude decreases. For the non-degenerate case (idler and signal photons in different wavelengths), this experimental scheme enables one to measure spectral or spatial phase property of materials beyond the detection limit of idler detector that might have lower quantum efficiency compared to the signal detector usually operating in the visible or near infrared region. This is the principle used in recent quantum imaging, where phase object is illuminated with photons that are ultimately not detected and the detected photons have not directly interacted with the sample^21, 22^. Similarly, in an IR spectroscopy measurements, instead of detecting IR photons which interacted directly with the sample, the conjugate visible photons were detected instead that carried the information of the IR absorption^23^. From a spectroscopy viewpoint, the interferometer involving two SPDC crystals generates path-entangled photon pairs and the photon pair from the first crystal can be considered as a radiation source. The idler photon generated from NL1, *i*_1_, is allowed to interact with the material of interest (Fig. 1b), but the conjugate signal photon *s*_1_ is in a second-order interference with a reference signal beam *s*_2_ generated from NL2. Thus, the one-photon interference between *s*_1_ and *s*_2_ reflects the degree of coherence between the conjugate *i*_1_ and *i*_2_ beams and it is modulated by the partially transmitting slab of material, i.e., the optical sample (OS) or beam splitter.

Now, let us consider the triple-SPDC configuration with three SPDC crystals aligned in a cascading geometry (Fig. 1c), where two partially reflecting planar materials (or dielectric slabs), OS1 and OS2, form a resonator in which optical sample cells are oriented such that surfaces are normal to the incident idler beams. Here, we show that a quantum optical measurement of OS1 under interaction with the idler beam from the radiation source, NL1, is made possible through a second-order interference measurement of the two signal beams from NL2 and NL3. This is highly counter-intuitive because none of the idler photons, *i*_1_, *i*_*2*_, and *i*_3_, is detected and the signal photons *s*_2_ and *s*_3_ are seemingly not in direct correlation with the *i*_1_ photon from NL1. This is achieved by taking full advantage of the quantum nature of *both* tripartite entanglement and vacuum modulation; the same cannot be said about the double-SPDC configuration or the ordinary triple-SPDC configuration proposed recently^24, 25^ (see Supplementary Information).

## Induced Coherence via vacuum indistinguishability

According to the theory of SPDC^26, 27^, the quantum state of the down-converted signal and idler photon pair generated from classical pump fields in the scheme illustrated in Fig. 1, is described by the interaction Hamiltonian $H=\sum _{j}\hslash g{A}_{{p}_{j}}({\omega }_{p},t){\hat{a}}_{{s}_{j}}^{\dagger }{\hat{a}}_{{i}_{j}}^{\dagger }({\omega }_{i})+h.c.$ with phase matching conditions *ω*_*p*_ = *ω*_*s*_ + *ω*_*i*_ and ${\overrightarrow{k}}_{p}={\overrightarrow{k}}_{s}+{\overrightarrow{k}}_{i}$, where ${\hat{a}}_{{s}_{j}({i}_{j})}$ is the annihilation operator of the signal (idler) field on NL*j*. The time-dependent state, when it is initially in a *vacuum* state |*vac*〉, is given by $|{\psi }_{0}(t)\rangle ={e}^{-i\int d{t}^{{\rm{^{\prime} }}}H({t}^{{\rm{^{\prime} }}})/\hslash }|vac\rangle$ and approximated in the weak coupling regime as

1 $$|{\psi }_{0}(t)\rangle \approx |vac\rangle +\sum _{j}\,{g}_{j}\iint d{\omega }_{s}d{\omega }_{i}{F}_{s}({\omega }_{s}){F}_{i}({\omega }_{i}){A}_{{p}_{j}}({\omega }_{p},t+{\tau }_{j}){|{\omega }_{s}\rangle }_{{s}_{j}}{|{\omega }_{i}\rangle }_{{i}_{j}},$$

where *g*_*j*_ is the coupling constant, *F*_*s*(*i*)_(*ω*_*s*(*i*)_) is the filter spectral distribution, ${A}_{{p}_{j}}({\omega }_{p},t)$ is the classical amplitude of the pump beam on NL*j*, and $|{\omega }_{s}\rangle$ ($|{\omega }_{i}\rangle$) is the single photon state in signal (idler) mode at frequency *ω*_*s*_ (*ω*_*i*_). The signal (idler) electric field operator is given as ${E}_{{k}_{j}}^{\dagger }(t)\propto \int d{\omega }_{k}{\hat{a}}_{{k}_{j}}({\omega }_{k}){e}^{-i{\omega }_{k}t}(k\in s,i)$. For the sake of clarity, we shall consider monochromatic fields here (see Supplementary Information for detailed calculation results for multimode cases).

As the proposed triple-SPDC scheme in Fig. 1c shows two partially transmitting slabs referred to as optical samples, OS1 and OS2, are placed on the idler beam path and the three idler beams, (*i*_1_, *i*_2_ and *i*_3_), are perfectly aligned. The OS1 is used to modulate the degree of indistinguishability between *i*_1_ and *i*_2_ beams, which, in turn, affects the degree of coherence between *s*_1_ and *s*_2_ beams at detector D12. The OS2 changes the degree of indistinguishability among the three idler beams but also among the two *vacuum* fields, denoted as *i*_01_ and *i*_02_, at the unused ports of the beam splitters, OS1 and OS2, that together form a planar resonator. In fact, we shall show that a resonator effect determining the complex amplitude of round-trip attenuation factor is evident in the second-order interference at D13. Since the presence of two slabs further modifies modal structures of transverse vacuum field along the idler beam pathway, a second-order interference between two signal beams *s*_2_ and *s*_3_, which is mediated by quantum vacuum field, can be observed at D23. This is the critical difference in our scheme compared to the other triple-SPDC schemes investigated before^24, 25^. The relations among the annihilation operators (amplitudes) of input modes (${\hat{a}}_{{i}_{1}},{\hat{a}}_{{i}_{02}}$), internal cavity modes $({\hat{a}}_{{i}_{2}},{\hat{a}}_{{i}_{01}})$, and output modes $({\hat{a}}_{{i}_{3}},{\hat{a}}_{{i}_{1}^{{\rm{^{\prime} }}}})$ are related to one another as $$(\begin{array}{c}{\hat{a}}_{{i}_{2}}\\ {\hat{a}}_{{i}_{01}}\end{array})=B(\begin{array}{c}{\hat{a}}_{{i}_{1}}\\ {\hat{a}}_{{i}_{02}}\end{array})$$ and $$(\begin{array}{c}{\hat{a}}_{{i}_{3}}\\ {\hat{a}}_{{i}_{1}^{{\rm{^{\prime} }}}}\end{array})=C(\begin{array}{c}{\hat{a}}_{{i}_{1}}\\ {\hat{a}}_{{i}_{02}}\end{array})$$ ^28^, where *i*_01_(*i*_02_) is the *vacuum* mode at the unused port of OS1(OS2) (Fig. 1c). The transfer matrices of OS1 and OS2 are given by^28^;

2 $$B=\frac{1}{1-{R}_{1}{R}_{2}{e}^{i2\delta }}(\begin{array}{cc}{T}_{1} & {T}_{2}{R}_{1}{e}^{i2\delta }\\ {T}_{1}{R}_{2}{e}^{i2\delta } & {T}_{2}\end{array})\,\,\,\,{\rm{and}}$$

3 $$C=(\begin{array}{cc}\frac{{T}_{2}{T}_{1}}{1-{R}_{1}{R}_{2}{e}^{i2\delta }} & {R}_{2}+\frac{{T}_{2}^{2}{R}_{1}{e}^{i2\delta }}{1-{R}_{1}{R}_{2}{e}^{i2\delta }}\\ {R}_{1}+\frac{{T}_{1}^{2}{R}_{2}{e}^{i2\delta }}{1-{R}_{1}{R}_{2}{e}^{i2\delta }} & \frac{{T}_{1}{T}_{2}}{1-{R}_{1}{R}_{2}{e}^{i2\delta }}\end{array}),$$

where transmission (reflection) coefficients of symmetric dielectric slabs OS1 and OS2 are *T*_1_ and *T*_2_ (*R*_1_ and *R*_2_), and *δ* denotes the phase gained from the beam propagation between OS1 and OS2. Here, we neglect the phase shifts gained by beam propagation through nonlinear crystals, since they do not affect the main results. If we assume ${R}_{j}=i\sqrt{1-{T}_{j}^{2}}(j=1,2)$ for simplicity, the quantum state for the field in Fig. 1c is given by

4 $$\begin{array}{ccc}|{\psi }_{3}(t)\rangle  & \approx  & |vac\rangle +({g}_{1}{A}_{{p}_{1}}(t)|{\omega }_{s},0,0\rangle +{g}_{2}{A}_{{p}_{2}}(t+{\tau }_{0}){e}^{-i{\phi }_{0}}\frac{{B}_{11}^{\ast }}{\sqrt{{\rm{N}}}}|0,{\omega }_{s},0\rangle \\  &  & {+{g}_{3}{A}_{{p}_{3}}(t+{\tau }_{1}){e}^{-i{\phi }_{1}}{C}_{11}^{\ast }|0,0,{\omega }_{s}\rangle )}_{{s}_{1},{s}_{2},{s}_{3}}{|{\omega }_{i},0\rangle }_{{i}_{1},{i}_{02}}\\  &  & +\,{({g}_{2}{A}_{{p}_{2}}(t+{\tau }_{0}){e}^{-i{\phi }_{0}}\frac{{B}_{12}^{\ast }}{\sqrt{{\rm{N}}}}|0,{\omega }_{s},0\rangle +{g}_{3}{A}_{{p}_{3}}(t+{\tau }_{1}){e}^{-i{\phi }_{1}}{C}_{12}^{\ast }|0,0,{\omega }_{s}\rangle )}_{{s}_{1},{s}_{2},{s}_{3}}{|0,{\omega }_{i}\rangle }_{{i}_{1},{i}_{02}}\end{array}$$

where *φ*_0_(*φ*_1_) and *τ*_0_(*τ*_1_) are the phase factor and time delay gained, respectively, when it propagates from NL1 to NL2 (from NL2 to NL3), and N is the normalization factor defined as ${\rm{N}}={|{B}_{11}|}^{2}+{|{B}_{12}|}^{2}$. We note that equation (4) is obtained via the transformation ${|{\omega }_{i}\rangle }_{{i}_{2}}={a}_{{i}_{2}}^{\dagger }|vac\rangle =({B}_{11}^{\ast }{|{\omega }_{i}\rangle }_{{i}_{1}}+{B}_{12}^{\ast }{|{\omega }_{i}\rangle }_{{i}_{02}})/\sqrt{{\rm{N}}}$ and ${|{\omega }_{i}\rangle }_{{i}_{3}}={a}_{{i}_{3}}^{\dagger }|vac\rangle =({C}_{11}^{\ast }{|{\omega }_{i}\rangle }_{{i}_{1}}+$ ${C}_{12}^{\ast }{|{\omega }_{i}\rangle }_{{i}_{02}})$. The density matrix of signal photon state is obtained by tracing out idler states, *ρ*_*s*_ = Tr_*i*_|*ψ*_3_〉〈*ψ*_3_|, and can be written as follows:

5 $$\begin{array}{rcl}{\rho }_{s} & = & |vac\rangle \langle vac|+{|{g}_{1}|}^{2}{I}_{1}(t)|{\omega }_{1},0,0\rangle \langle {\omega }_{1},0,0|+{|{g}_{2}|}^{2}{I}_{2}(t+{\tau }_{0})|0,{\omega }_{2},0\rangle \langle 0,{\omega }_{2},0|\\  &  & +\,{|{g}_{3}|}^{2}{I}_{3}(t+{\tau }_{1})|0,0,{\omega }_{3}\rangle \langle 0,0,{\omega }_{3}|\\  &  & +\,{g}_{1}{g}_{2}^{\ast }{A}_{{p}_{1}}(t){A}_{{p}_{2}}^{\ast }(t+{\tau }_{0})\frac{{B}_{11}^{\ast }}{\sqrt{{\rm{N}}}}|{\omega }_{1},0,0\rangle \langle 0,{\omega }_{2},0|\\  &  & +\,{g}_{1}{g}_{3}^{\ast }{A}_{{p}_{1}}(t){A}_{{p}_{3}}^{\ast }(t+{\tau }_{1}){C}_{11}|{\omega }_{1},0,0\rangle \langle 0,0,{\omega }_{3}|\\  &  & +\,{g}_{2}{g}_{3}^{\ast }{A}_{{p}_{2}}(t+{\tau }_{0}){A}_{{p}_{3}}^{\ast }(t+{\tau }_{1})\frac{{B}_{11}^{\ast }{C}_{11}+{B}_{12}^{\ast }{C}_{12}}{\sqrt{{\rm{N}}}}|0,{\omega }_{2},0\rangle \langle 0,0,{\omega }_{3}|+h.c.\end{array}$$

The three second-order interference fringe visibilities detected at D12, D13, and D23 thus can be obtained from the photon counting rate defined as ${R}_{s,ij}\propto \langle {\psi }_{3}(t)|{E}_{s,{\rm{D}}ij}^{-}{E}_{s,{\rm{D}}ij}^{\dagger }|{\psi }_{3}(t)\rangle$ and are given by

6 $$\begin{array}{ccc}{V}_{12} & = & \frac{2|{g}_{1}^{\ast }{g}_{2}|\langle {A}_{{p}_{1}}^{\ast }(t){A}_{{p}_{2}}(t+{\tau }_{0})\rangle }{({|{g}_{1}|}^{2}\langle {I}_{{p}_{1}}(t)\rangle +{|{g}_{2}|}^{2}\langle {I}_{{p}_{2}}(t+{\tau }_{0})\rangle )}|\frac{{B}_{11}}{\sqrt{{\rm{N}}}}|,\\ {V}_{13} & = & \frac{2|{g}_{1}^{\ast }{g}_{3}|\langle {A}_{{p}_{1}}^{\ast }(t){A}_{{p}_{3}}(t+{\tau }_{1})\rangle }{({|{g}_{1}|}^{2}\langle {I}_{{p}_{1}}(t)\rangle +{|{g}_{3}|}^{2}\langle {I}_{{p}_{3}}(t+{\tau }_{1})\rangle )}|{C}_{11}|,\,{\rm{a}}{\rm{n}}{\rm{d}}\\ {V}_{23} & = & \frac{2|{g}_{2}^{\ast }{g}_{3}|\langle {A}_{{p}_{2}}^{\ast }(t+{\tau }_{0}){A}_{{p}_{3}}(t+{\tau }_{1})\rangle |}{({|{g}_{2}|}^{2}\langle {I}_{{p}_{2}}(t+{\tau }_{0})\rangle +{|{g}_{3}|}^{2}\langle {I}_{{p}_{3}}(t+{\tau }_{1})\rangle )}|\frac{{B}_{11}^{\ast }{C}_{11}+{B}_{12}^{\ast }{C}_{12}}{\sqrt{{\rm{N}}}}|,\end{array}$$

where ${I}_{{p}_{j}}(t)={|{A}_{{p}_{j}}(t)|}^{2}(j\in 1,2,3)$. For the identical pump beams at three crystals, ${|{g}_{1}|}^{2}\langle {I}_{{p}_{1}}(t)\rangle ={|{g}_{2}|}^{2}\langle {I}_{{p}_{2}}(t+{\tau }_{0})\rangle \,=$ ${|{g}_{3}|}^{2}\langle {I}_{{p}_{3}}(t+{\tau }_{1})\rangle$, and for perfect temporal and spatial overlaps of idler modes, $\tfrac{\langle {A}_{{p}_{1}}(t){A}_{{p}_{2}}(t+{\tau }_{0})\rangle }{\langle {A}_{{p}_{1}}(t)\rangle \langle {A}_{{p}_{2}}(t+{\tau }_{0})\rangle }\,=1,$ $\tfrac{\langle {A}_{{p}_{1}}(t){A}_{{p}_{3}}(t+{\tau }_{1})\rangle }{\langle {A}_{{p}_{1}}(t)\rangle \langle {A}_{{p}_{3}}(t+{\tau }_{1})\rangle }=1,\tfrac{\langle {A}_{{p}_{2}}(t+{\tau }_{0}){A}_{{p}_{3}}(t+{\tau }_{1})\rangle }{\langle {A}_{{p}_{2}}(t+{\tau }_{0})\rangle \langle {A}_{3}(t+{\tau }_{1})\rangle }=1$, respectively.

The visibilities in equation (6) at each detector are simplified as

7 $$\begin{array}{rcl}{V}_{12} & = & \frac{|{T}_{1}|}{\sqrt{{|{T}_{1}|}^{2}+{|{T}_{2}|}^{2}-{|{T}_{1}|}^{2}{|{T}_{2}|}^{2}}},\\ {V}_{13} & = & \frac{|{T}_{1}\,{T}_{2}|}{|1+\sqrt{1-{T}_{1}^{2}}\sqrt{1-{T}_{2}^{2}}{e}^{i2\delta }|},\,{\rm{and}}\\ {V}_{23} & = & \frac{|{T}_{2}|}{\sqrt{{|{T}_{1}|}^{2}+{|{T}_{2}|}^{2}-{|{T}_{1}|}^{2}{|{T}_{2}|}^{2}}},\end{array}$$

where *T*_1_ and *T*_2_ are not to be zero simultaneously. We note that the fringe visibility at D13 depends on the cavity resonance whereas the other two visibilities at D12 and D23 do not depend on the cavity phase *δ* due to the cancellation of phase-dependent term in the normalization factor. We find that the visibility of each interference fringe of a pair of *signal* beams is determined by the degree of overlap between the corresponding pair of *idler* states, i.e., ${V}_{12}=|{}_{{i}_{1}}\langle {\omega }_{i}|{\omega }_{i}\rangle _{{i}_{2}}|$, ${V}_{13}=|{}_{{i}_{1}}\langle {\omega }_{i}|{\omega }_{i}\rangle _{{i}_{3}}|$, and ${V}_{23}=|{}_{{i}_{2}}\langle {\omega }_{i}|{\omega }_{i}\rangle _{{i}_{3}}|$ as shown in equation (7). Interestingly, the indistinguishability of *vacuum* modes, *i*_01_ and *i*_02_, at the two *vacuum* (unused) ports of OS1 and OS2, $|{}_{{i}_{01}}\langle {\omega }_{i}|{\omega }_{i}\rangle _{{i}_{02}}|$, is exactly the same with $|{}_{{i}_{2}}\langle {\omega }_{i}|{\omega }_{i}\rangle _{{i}_{3}}|$.

## Discussions

To understand the underlying physics of this triple-SPDC scheme, it is helpful to consider limiting cases first. If OS2 is removed, we have *T*_2_ = 1 and *R*_2_ = 0, *i*_2_ and *i*_3_ beams are fully indistinguishable due to the perfect alignment of the corresponding idler beams. On the other hand, the degrees of indistinguishability between *i*_1_ and *i*_2_ and between *i*_1_ and *i*_3_ are modulated by the factor |*T*_1_| as *V*_12_ = *V*_13_ = |*T*_1_| and *V*_23_ = 1. Similarly, if OS1 is removed, i.e., *T*_1_ = 1 and *R*_1_ = 0, we find *V*_12_ = 1 and *V*_13_ = *V*_23_ = |*T*_2_|. These results are identical to the case of double-SPDC scheme (see Methods equation (9)) since the resonator and vacuum indistinguishability effects vanish in these cases.

The other limiting case is when one of the OS1 and OS2 is a perfect two-way mirror with unit reflectivity, i.e., either *T*_1_ = 0(*R*_1_ = *i*) or *T*_2_ = 0(*R*_2_ = *i*). When *T*_1_ = 0 and *R*_1_ = *i*, one can immediately find *V*_12_ = *V*_13_ = 0 from Eq. (7), which results from the complete destruction of coherences between *i*_1_ and *i*_2_ and between *i*_1_ and *i*_3_ because the idler 1 is totally reflected by the OS1. However, the second-order interference between *s*_2_ and *s*_3_ does not vanish and even becomes unity, (*V*_23_ = 1), regardless of the transmissivity (*T*_2_) of OS2; this is because *i*_2_ and *i*_3_ are maximally indistinguishable, that is ${}_{{i}_{2}}\langle {\omega }_{i}|{{\omega }_{i}\rangle }_{{i}_{3}}=1$, in the presence of such perfectly reflecting two-way mirror at OS1. Similarly, if a two-way planar mirror replaces the OS2, then *T*_2_ = 0 and *R*_2_ = *i*, so indistinguishability between *i*_1_(*i*_2_) and *i*_3_ will be destroyed; this, in turn, makes the second-order interference between *s*_1_(*s*_2_) and *s*_3_ to vanish, i.e., *V*_13_ = *V*_23_ = 0, and *V*_12_ = 1. We note that the second-order interference visibility is always unity and independent of the transmissivity of optical sample with one side of resonator having the perfect reflectivity.

**Figure 2:**
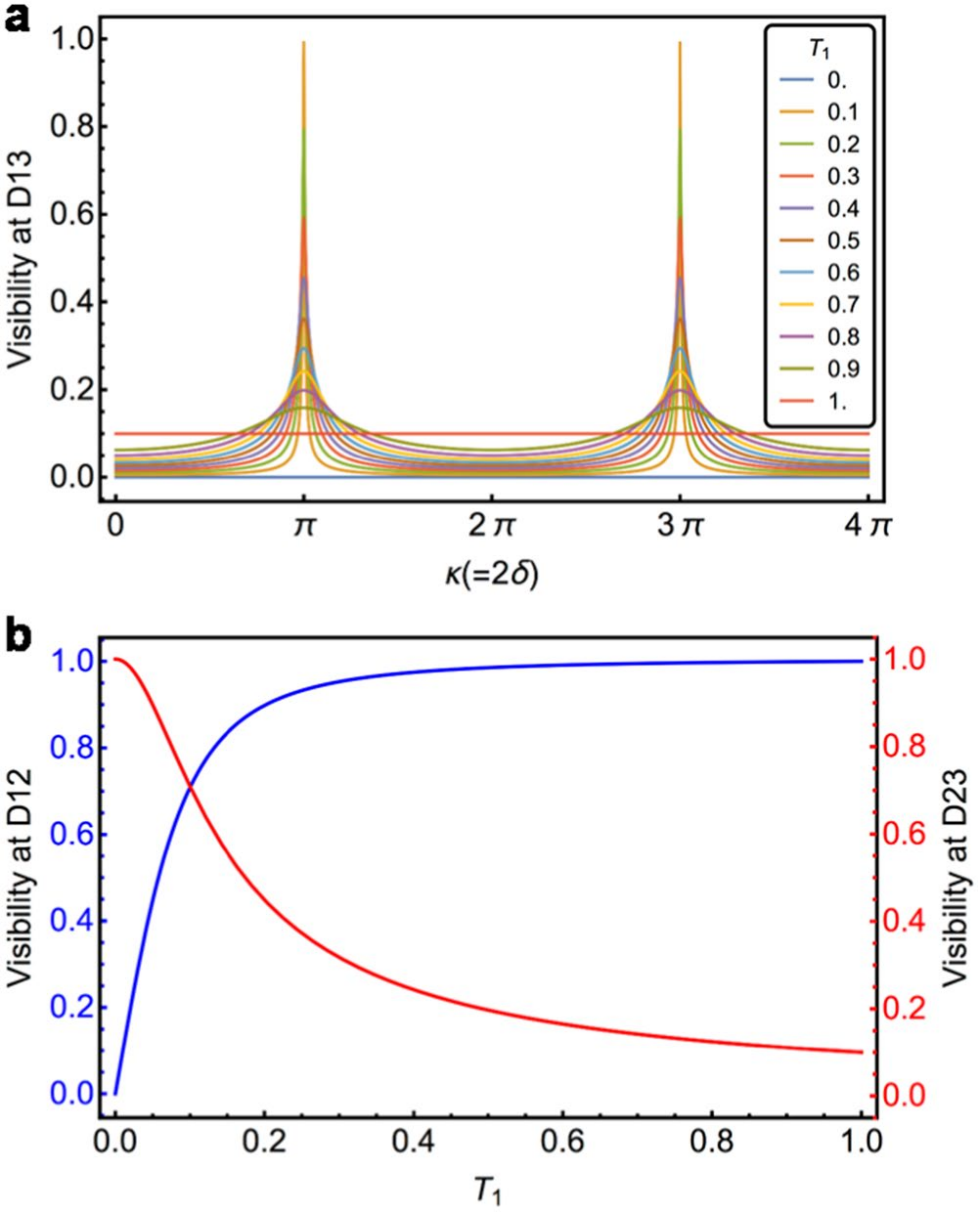
Numerical calculation results. (**a**) Visibility of interference fringe at D13, *V*_13_, with respect to amplitude phase gained by a round trip in cavity, *κ* = 2*δ*, for varying transmissivity (*T*_1_) of OS1 with *T*_2_ = 0.1. We note that transmission becomes 1 on resonance for *T*_2_ = *T*_1_ due to the cavity input-output relation. (**b**) Visibility of interference fringe at D12 (blue) and D23 (red), *V*_23_ and *V*_12_, with respect to amplitude transmissivity of OS1, when *T*_2_ = 0.1.

A more interesting and highly counter-intuitive case is when both OS1 and OS2 are neither perfectly reflecting nor transmitting materials. The degree of coherence between *s*_1_ and *s*_3_ can be measured by detecting the second-order interference at D13 as shown in Fig. 2a and the visibility *V*_13_ is found to be dependent on the distinguishability of the input and output idler modes of the cavity formed by OS1 and OS2. Surprisingly, the visibility at D23 does depend on *T*_1_ (see equation (7)), which means that the optical property of a target sample at OS1 can be extracted from second-order interference measurements involving *s*_2_ and *s*_3_. For example, when *T*_2_ = 0.1, the visibility at D23 becomes larger as the transparency of sample OS1 decreases, i.e., ${T}_{1}\to 0\,({R}_{1}\to i)$ (see Fig. 2b). This is a counter-intuitive result, because it indicates that an increasing disturbance by OS1 (decreasing transmissivity of OS1) causes the coherence between *s*_2_ and *s*_3_ to increase. However, this becomes understandable if it is noted that the OS1 with *T*_1_ ≠ 1 plays not only the role of a decoherence material but also as a mirror forming a resonator together with OS2. For a finite *T*_2_, the visibility at D23 approaches to its maximum value of 1 at *T*_1_ = 0 (see Supplementary Information). Thus, a coherence between *s*_2_ and *s*_3_ is critically induced when the *vacuum* mode at the unused port of OS1 becomes indistinguishable with OS2 in place to form a resonator due to the cavity effect. In particular, pathway distinguishabilities of the three idler beams are effectively erased by forming an optical resonator with NL2 inside on the idler beam pathway by two slabs. Thereby, a full quantum optical measurement of spectral (or phase) property of the OS1 is possible by detecting the second-order interference between *s*_2_ and *s*_3_ that are not affected by the SPDC process at NL1 located outside of the resonator cavity.

To gain a deeper insight into the proposed triple-SPDC quantum optical measurement, it is necessary to understand how OS2, a planar boundary object, alters both idler and vacuum mode structures. Coherences of the three signal modes are induced by two different mechanisms. In equation (4), the second term describes the induced coherence of signal beams *s*_1_, *s*_2_, and *s*_3_ via indistinguishabilities of their entangled idler states *i*_1_, *i*_2_, and *i*_3_. It is the third term in equation (4) that describes a further induced coherence between *s*_2_ and *s*_3_ via the indistinguishability of *i*_2_, *i*_3_, and *i*_02_. Note that, when the idler beams are perfectly aligned and OS1 and OS2 are absent, the one-photon signal state is nothing but a maximally superposed state of three signal modes, i.e., ${|\psi \rangle }_{s} \sim {(|{\omega }_{s},0,0\rangle +|0,{\omega }_{s},0\rangle +|0,0,{\omega }_{s}\rangle )}_{{s}_{1},{s}_{2},{s}_{3}}$, which is a *W*-type path-entangled single photon state. For a W-type entanglement as opposed to a GHZ (Greenberger-Horne-Zeilinger) state, it is well known that a disturbance on one of the three modes does not affect the remaining two-mode entanglement^29^. Here, even in the case that one of idler beams is disturbed by either OS1 or OS2, the remaining idler beams can still be indistinguishable leading to the coherence between the corresponding signal modes. In the presence of OS1, an additional insertion of OS2 between NL2 and NL3 causes two intriguing effects: (i) it reduces the degree of coherence between *i*_2_ and *i*_3_ and (ii) it changes the modal structure of the *vacuum* since the partially transparent OS2 forms an optical cavity with OS1 and makes the vacuum modes, *i*_02_ and *i*_01_, at unused ports of OS1 and OS2 indistinguishable. The latter is the essential and unique feature of our proposed experimental scheme for detecting *T*_1_ by means of measuring the fringe visibility at D23.

### Experimental feasibility

Now, let us discuss about the critical issues in experimental feasibility. First, the spectral overlap of all idler beams between input mode *i*_1_, inside cavity mode *i*_2_, and output mode *i*_3_ is required. If we consider the multimode spectral distribution of single photons generated from SPDC, the spectral overlap between input, internal cavity, and output modes would be decreased. Thus, all narrowband single photons are preferred to increase spectral overlap. Second, temporal overlaps of all idler beams are required. The time delay due to the round trip of cavity mode will cause temporal overlaps mismatches of idler photons. This can be solved by either using a phase-coherent optical frequency comb^30^ for the pump beam to ensure long coherence time or compensating delay by putting Fabry-Pérot cavities on an appropriate path in the interferometer (see Supplementary Information). Third, almost perfectly reflective material along longitudinal axis is required. Here, we treated OS1 and OS2 as lossless beam splitters. However, one should be careful about treating absorption of light by a given material for practical applications of our proposed triple-SPDC scheme to quantum spectroscopy. For such circumstances, our theoretical description needs to be generalized to include the input-output relationships in a lossy beam splitter by treating the light absorption as a loss in field amplitude^31^. Fourth, the second-order interference measurement is usually affected intrinsic dark noise, whereas the coincident counting shows zero dark noise in principle. Our scheme would work well for the case of weak transparent resonance signal, e.g., electromagnetically induced transparency^32^, because small *T*_1_ sample probed by *i*_1_ would give a high visibility for the second-order interference detection of signals *s*_2_ and *s*_3_ at D23.

## Conclusion

The single-SPDC scheme for spectroscopic or imaging application requires coincidence detections of both signal idler photons, where either one of the two interacts with absorptive material or phase object, via two-photon interference measurement or fourth-order (in the field) interference. In contrast, the double-SPDC scheme needs no detection of idler photons, but it still requires a measurement of a second-order interference between conjugate signal beams from the radiation source. In this Letter, we have shown that the proposed triple-SPDC scheme requires detections of neither idler photons interacting with material (or phase object) nor their conjugate signal photons, due to the indistinguishability of the *vacuum* fields. This scheme is the first of its kind that shows how vacuum field indistiguishability plays a role in spectroscopy (or imaging) and clearly differentiates from the previous quantum spectroscopy or imaging studies with just one or two SPDC crystals. We anticipate that the proposed triple-SPDC scheme involving three down-converters arranged in a cascading geometry will be of great interest and use for quantum spectroscopy, quantum imaging, and potential applications to quantum information technology with tripartite entangled state.

## Methods

### Induced Coherence via idler indistinguishability

For a double-SPDC experiment with two NL crystals arranged in a cascading configuration as depicted in Fig. 1b, two idler paths are indistinguishable as *i*_1_ and *i*_2_ are perfectly aligned. At the optical sample (OS) placed in the idler beam path, the amplitudes of *i*_1_ and *i*_2_ are related to each other: ${\hat{a}}_{{i}_{2}}=T{\hat{a}}_{{i}_{1}}+R^{\prime} {\hat{a}}_{{i}_{0}}$ ^33, 34^, where *T*(*R*) and *T*′(*R*′) with |*T*|^2^ + |*R*′|^2^ = 1, are the transmission (reflection) coefficients of the front and rear sides of the OS, respectively, ${\hat{a}}_{{i}_{1}}$ and ${\hat{a}}_{{i}_{2}}$ are the annihilation operators of *i*_1_ and *i*_2_, and ${\hat{a}}_{{i}_{0}}$ is that of the *vacuum* field at the unused port of OS. The initial path-entangled state is then disturbed by the OS (e.g., a beam splitter), so that the time-dependent state becomes

8 $$\begin{array}{ccc}|{\psi }_{2}(t)\rangle  & \approx  & |vac\rangle +({g}_{1}{A}_{{p}_{1}}(t){|{\omega }_{s},0\rangle }_{{s}_{1},{s}_{2}}+{g}_{2}{A}_{{p}_{2}}(t+{\tau }_{0}){e}^{-i{\phi }_{0}}{T}^{\ast }{|0,{\omega }_{s}\rangle }_{{s}_{1},{s}_{2}}){|{\omega }_{i},0\rangle }_{{i}_{1},{i}_{0}}\\  &  & +\,{g}_{2}{A}_{{p}_{2}}(t+{\tau }_{0}){e}^{-i{\phi }_{0}}{R}^{{\rm{^{\prime} }}\ast }{|0,{\omega }_{s}\rangle }_{{s}_{1},{s}_{2}}{|0,{\omega }_{i}\rangle }_{{i}_{1},{i}_{0}},\end{array}$$

where *φ*_0_ is the phase factor gained due to the time delay *τ*_0_ by beam propagation from NL1 to NL2. As shown in Eq. (8), one photon coherence of a signal photon is induced as much as the transmissivity *T*. One-photon interference or second-order interference for two signal beams is to measure average photon count rate *R*_*s*_ at the detector ${{\rm{D}}}_{s}^{{\rm{^{\prime} }}}$ and it is related to the corresponding second-order correlation function ${R}_{s}\sim \langle {\psi }_{2}(t)|{E}_{s,{{\rm{D}}}_{s}^{{\rm{^{\prime} }}}}^{-}{E}_{s,{{\rm{D}}}_{s}^{{\rm{^{\prime} }}}}^{\dagger }|{\psi }_{2}(t)\rangle$ where ${E}_{s,{{\rm{D}}}_{s}^{{\rm{^{\prime} }}}}^{\dagger }={\hat{a}}_{{s}_{1}}{e}^{i{\phi }_{1}}+{\hat{a}}_{{s}_{2}}{e}^{i{\phi }_{2}}$ and *φ*_1_(*φ*_2_) is the phase gained by beam propagation from NL1 (NL2) to ${{\rm{D}}}_{s}^{{\rm{^{\prime} }}}$. Since the second-order coherence of signal beams is induced by the idler beam indistinguishability, the fringe visibility at ${{\rm{D}}}_{s}^{{\rm{^{\prime} }}}$ is linearly proportional to the material transmissivity |*T*| as

9 $${V}_{s,{{\rm{D}}}_{s}^{{\rm{^{\prime} }}}}=|T|\frac{2|{g}_{1}^{\ast }{g}_{2}|\langle {A}_{{p}_{1}}^{\ast }(t){A}_{{p}_{2}}(t+{\tau }_{0})\rangle }{{|{g}_{1}|}^{2}{\langle {A}_{{p}_{1}}(t)\rangle }^{2}+{|{g}_{2}|}^{2}{\langle {A}_{{p}_{2}}(t+{\tau }_{0})\rangle }^{2}}.$$

Thus, one can measure the spectroscopic property, i.e., the frequency-dependent transmissivity *T*(*ω*_*i*_), of the material under interaction with the idler *i*_1_ via detection of the second-order interference of its conjugate signal *s*_1_ with the reference signal *s*_2_, by tuning the pump frequency *ω*_*p*_. This is the essential feature of the previous quantum spectroscopy and imaging that makes use of the double-SPDC scheme^23, 35^.

## Electronic supplementary material

Supplementary Information
