# Supplementary material for: Quantum optical measurements with undetected photons through vacuum field indistinguishability
Source: Sci Rep. 2017 Jul 26;7:6558. doi: 10.1038/s41598-017-06800-0 (PMC5529466; doi:10.1038/s41598-017-06800-0)
Supplement: Supplementary file 1 — Supplementary Information [file 41598_2017_6800_MOESM1_ESM.pdf]

Supplementary Information for

Quantum optical measurements with undetected photons through vacuum field indistinguishability

Sun Kyung Lee[1], Tai Hyun Yoon[1,2], and Minhaeng Cho[1,3*]

[1]*Center for Molecular Spectroscopy and Dynamics, Institute for Basic Science, Seoul 02841, Korea*

[2]*Department of Physics, Korea University, Seoul 02841, Korea*

[3]*Department of Chemistry, Korea University, Seoul 02841, Korea*

*E-mail: mcho@korea.ac.kr

Contents

I. Fundamental quantum theory of spontaneous parametric down conversion (SPDC)

II. Cascading configuration with two SPDC crystals

III. Cascading configuration with three SPDC crystals

A. Distinguishable quantum vacuum fields

B. Indistinguishable quantum vacuum fields: Optical resonator configuration

IV. Experimental Feasibility: A Few Critical Issues

Supplementary References

Supplementary Figures

I. Fundamental Quantum Theory of Spontaneous Parametric Down Conversion (SPDC)

In this section, we present a brief description on the quantum theory of SPDC for the sake of completeness (see recent reviews, references S1 and S2, for detailed and complete theoretical descriptions). When an electric field $\mathbf{E}(\mathbf{r},t)$ propagates through a nonlinear and non-centrosymmetric medium, the i th component of the electric polarization of the optical medium is approximately given by

$$P_i(\mathbf{r},t) = \varepsilon_0 \int_0^\infty dt' \chi_{ij}^{(1)}(t') E_j(\mathbf{r},t-t') + \int_0^\infty dt' \int_0^\infty dt'' \chi_{ijk}^{(2)}(t',t'') E_j(\mathbf{r},t-t') E_k(\mathbf{r},t-t''), \quad \text{(S1)}$$

where $E_j(\mathbf{r},t)$ is the j th component of the electric field vector and $\chi^{(1)}$ and $\chi^{(2)}$ are the first and second order susceptibility tensors, respectively. In Eq. (S1), all the higher-order contributions to the polarization are ignored. Throughout this Supplementary Information, the Einstein summation convention will be used. The electromagnetic field Hamiltonian in the medium of volume V is given as

$$H(t) = \frac{1}{2}\int_V dr [\mathbf{D}(\mathbf{r},t)\cdot\mathbf{E}(\mathbf{r},t) + \mathbf{B}(\mathbf{r},t)\cdot\mathbf{H}(\mathbf{r},t)], \quad \text{(S2)}$$

where $\mathbf{D}$ $(=\varepsilon_0\mathbf{E}(\mathbf{r},t)+\mathbf{P}(\mathbf{r},t))$, $\mathbf{B}$, and $\mathbf{H}$ are the displacement vector, the magnetic induction, and the magnetic field, respectively. Inserting the polarization in Eq. (S1) into Eq. (S2), one can find the perturbation (nonlinear field-matter interaction) Hamiltonian,

$$\begin{aligned} H_I(t) &= \frac{1}{2}\int_V dr\, \mathbf{E}(\mathbf{r},t)\cdot\mathbf{P}_{nl}(\mathbf{r},t) \\ &= \frac{1}{2}\int_V d\mathbf{r} \int_0^\infty dt' \int_0^\infty dt''\, \chi_{ijk}^{(2)}(t',t'') E_i(r,t) E_j(r,t-t') E_k(r,t-t''). \end{aligned} \quad \text{(S3)}$$

Here, $\mathbf{P}_{nl}(\mathbf{r},t)$ is the nonlinear component of the electric polarization. In the present work, we are interested in the nonlinear (second-order parametric down conversion) field-matter interaction in Eq. (S3) only.

To apply the quantization procedure, we expand the classical optical electric field, $\mathbf{E}(\mathbf{r},t)=\mathbf{E}^{+}(\mathbf{r},t)+\mathbf{E}^{-}(\mathbf{r},t)$, in terms of plane waves as

$$\mathbf{E}^{+}(\mathbf{r},t)=[\mathbf{E}^{-}(\mathbf{r},t)]^{*}=\frac{1}{\sqrt{\mathcal{V}}}\sum_{\mathbf{k},\sigma}e_{\mathbf{k},\sigma}\varepsilon_{\mathbf{k},\sigma}\alpha_{\mathbf{k},\boldsymbol{\sigma}}F(\omega)\exp[i(\mathbf{k}\cdot\mathbf{r}-\omega t)] \tag{S4}$$

where $\mathcal{V}$ is the field quantization volume, $F(\omega)$ is the transmission function for a filter placed in front of detector in a real experiment, and $\varepsilon_{\mathbf{k},\boldsymbol{\sigma}}=\sqrt{\hbar\omega(\mathbf{k},\sigma)/(2\varepsilon_0 n^2(\mathbf{k},\sigma))}$. Here, the linear refractive index of material is denoted as $n(\mathbf{k},\sigma)$, $\mathbf{k}$ is the wave vector, σ represents the two orthogonal components of the transverse plane wave, and $\alpha_{\mathbf{k},\sigma}$ is the mode amplitude. In Eq. (S4), the interference filter that is fully characterized by the transmission function is taken into consideration to realistically describe experiments with frequency filters and detectors used to select specific frequency components of signal, idler, and detected fields. Quantization of the electric field is to replace the mode amplitude in Eq. (S4) with the corresponding photon annihilation operator so that the corresponding electric field amplitude becomes a field operator, i.e.,

$$\mathbf{E}^{+}(\mathbf{r},t)=[\mathbf{E}^{-}(\mathbf{r},t)]^{\dagger}=\frac{1}{\sqrt{\mathcal{V}}}\sum_{\mathrm{k},\sigma}\mathbf{e}_{k,\sigma}\varepsilon_{k,\sigma}\hat{a}_{k,\sigma}F(\omega)\exp[i(\mathbf{k}\cdot\mathbf{r}-\omega t)]. \tag{S5}$$

Inserting this electric field operator into Eq. (S3), one finds that the interaction Hamiltonian operator describing SPDC is given as

$$\begin{aligned}\hat{H}_I &=\frac{1}{2\mathcal{V}^{3/2}}\sum_{\mathbf{k}_s,\sigma_s}\sum_{\mathbf{k}_i,\sigma_i}\sum_{\mathbf{k}_p,\sigma_p}g^{*}_{\mathbf{k}_s,\sigma_s}g^{*}_{\mathbf{k}_i,\sigma_i}g_{\mathbf{k}_p,\sigma_p}\hat{a}^{+}_{\mathbf{k}_s,\sigma_s}\hat{a}^{+}_{\mathbf{k}_i,\sigma_i}\hat{a}_{\mathbf{k}_p,\sigma_p}\chi_{ijk}(\mathbf{e}_{\mathbf{k}_s,\sigma_s})^{*}_i(\mathbf{e}_{\mathbf{k}_i,\sigma_i})^{*}_j(\mathbf{e}_{\mathbf{k}_p,\sigma_p})_k\\ &\quad\times\exp[i(\omega_s+\omega_i-\omega_p)t]\int_{\mathrm{V}}\exp[-i(\mathbf{k}_s+\mathbf{k}_i-\mathbf{k}_p)\cdot\mathbf{r}]d\mathbf{r}+h.c.\\ &=\frac{1}{2\mathcal{V}^{3/2}}\sum_{\mathbf{k}_s,\sigma_s}\sum_{\mathbf{k}_i,\sigma_i}\sum_{\mathbf{k}_p,\sigma_p}g^{*}_{\mathbf{k}_s,\sigma_s}g^{*}_{\mathbf{k}_i,\sigma_i}g_{\mathbf{k}_p,\sigma_p}\hat{a}^{+}_{\mathbf{k}_s,\sigma_s}\hat{a}^{+}_{\mathbf{k}_i,\sigma_i}\hat{a}_{\mathbf{k}_p,\sigma_p}\chi_{ijk}(\mathbf{e}_{\mathbf{k}_s,\sigma_s})^{*}_i(\mathbf{e}_{\mathbf{k}_i,\sigma_i})^{*}_j(\mathbf{e}_{\mathbf{k}_p,\sigma_p})_k\\ &\quad\times\exp[-i(\mathbf{k}_s+\mathbf{k}_i-\mathbf{k}_p)\cdot\mathbf{r}_0+i(\omega_s+\omega_i-\omega_p)t]\prod_m \mathrm{sinc}[(\mathbf{k}_s+\mathbf{k}_i-\mathbf{k}_p)_m l_m/2]+h.c.\end{aligned} \tag{S6}$$

where $g_{\mathbf{k}_j,\sigma_j} = i\varepsilon_{\mathbf{k}_j,\sigma_j} F(\omega(\mathbf{k}_j,\sigma_j))$, $l_m (m = x, y, z)$ is the length of the dielectric medium in the m th direction, $\mathbf{r}_0$ is the position vector of the center of the nonlinear crystal, and *h.c.* stands for Hermitian conjugate. The three fields involved in the SPDC process that is theoretically described by Eq. (S6) will be referred to as pump (p), signal (s), and idler (i).

The quantum state at time t, when the initial state at time zero is assumed to be $|\psi(0)\rangle$, is then given by

$$|\psi(t)\rangle = \hat{U}(t)|\psi(0)\rangle, \tag{S7}$$

where the time-evolution operator is

$$\hat{U}(t) = \exp\left(\frac{1}{i\hbar}\int_0^t d\tau \hat{H}_I(\tau)\right) = 1 + \left(\frac{1}{i\hbar}\int_0^t d\tau \hat{H}_I(\tau)\right) + \cdots. \tag{S8}$$

Typically, the pump field intensity is very weak and the field-matter interaction time is usually much shorter than the average time between down conversions. In this limit, the first two terms in the power series expansion of Eq. (S8) are sufficient enough to describe the quantum state at time t:

$$\begin{aligned}|\psi(t)\rangle = |vac\rangle + \frac{\mathrm{V}t}{2i\hbar\mathcal{V}^{3/2}} \sum_{\mathbf{k}_s,\sigma_s} \sum_{\mathbf{k}_i,\sigma_i} \sum_{\mathbf{k}_p,\sigma_p} g^*_{\mathbf{k}_s,\sigma_s} g^*_{\mathbf{k}_i,\sigma_i} g_{\mathbf{k}_p,\sigma_p} A_p(\mathbf{k}_p,\sigma_p) \chi_{ijk} (\mathbf{e}_{\mathbf{k}_s,\sigma_s})^*_i (\mathbf{e}_{\mathbf{k}_i,\sigma_i})^*_j (\mathbf{e}_{\mathbf{k}_p,\sigma_p})_k \\ \times \exp[-i(\mathbf{k}_s + \mathbf{k}_i - \mathbf{k}_p)\cdot \mathbf{r}_0 + i(\omega_s + \omega_i - \omega_p)t/2] \,\mathrm{sinc}[(\omega_s + \omega_i - \omega_p)t/2] \\ \times \prod_m \mathrm{sinc}[(\mathbf{k}_s + \mathbf{k}_i - \mathbf{k}_p)_m l_m / 2] |\mathbf{k}_s,\sigma_s\rangle |\mathbf{k}_i,\sigma_i\rangle,\end{aligned} \tag{S9}$$

where the single photon Fock states of signal and idler modes are denoted as $|\mathbf{k}_s,\sigma_s\rangle = \hat{a}^\dagger_{\mathbf{k}_s,\sigma_s}|vac\rangle$ and $|\mathbf{k}_i,\sigma_i\rangle = \hat{a}^\dagger_{\mathbf{k}_i,\sigma_i}|vac\rangle$, respectively. The quantum state of the pump beam used in a real experiment is essentially constant in SPDC and its depletion is negligible in comparison to its average intensity. Therefore, the pump beam can be safely treated classically and the annihilation operator of the pump field can be replaced with a classical

amplitude, $A_p(\mathbf{k}_p,\sigma_p)$. Then, invoking a series of reasonable approximations (see references S1-S3), the quantum state at time t in Eq. (S9) can be greatly simplified as

$$|\psi\rangle = |vac\rangle + \sum_{\sigma_s,\sigma_i}\int d\omega_s \int d\omega_i \int d\mathbf{q}_s \int d\mathbf{q}_i \Gamma_{\sigma_s\sigma_i}(\mathbf{q}_s,\mathbf{q}_i,\omega_s,\omega_i)|\mathbf{q}_s,\omega_s,\sigma_s\rangle|\mathbf{q}_i,\omega_i,\sigma_i\rangle, \quad \text{(S10)}$$

where $\Gamma_{\sigma_s\sigma_i} \approx C_{\sigma_s\sigma_i}F_s(\omega_s)F_i(\omega_i)A_p(\mathbf{q}_s+\mathbf{q}_i,\omega_s+\omega_i)\text{sinc}[(k_{sz}+k_{iz}-k_{pz})L/2]$ and $C_{\sigma_s\sigma_i}$ is a constant that is determined by the nonlinear susceptibility, $\chi^{(2)}$. In a real experiment, signal and/or idler beams are spatially filtered with dichroic mirrors and the corresponding detectors are placed far away from the SPDC NL crystal that can be considered as radiation source. In that case, the quantum state in Eq. (S10) can be simply written as

$$|\psi\rangle = |vac\rangle + C\int d\omega_s \int d\omega_i F_s(\omega_s)F_i(\omega_i)A_p(\omega_i+\omega_s)|\omega_s\rangle|\omega_i\rangle. \quad \text{(S11)}$$

In this limit, the electric field operator also takes a simple form (for $j=s$ and i):

$$\hat{E}_j^+(t) = E_0\int d\omega \hat{a}_j(\omega)\exp[-i\omega_j t]. \quad \text{(S12)}$$

Despite that a number of approximations were invoked to obtain both the quantum state (Eq. (S11) generated by a SPDC and the electric field operators (Eq. (S12)) of signal and idler photons, they properly describe most of the key features of temporal correlations between the quantum entangled signal and idler photons produced by SPDC. Often, such time correlation has been experimentally measured by detecting the coincidence counting rate that corresponds to the fourth-order (in the fields) correlation function or two-photon interference term:

$$R_c(t+\tau_i,t+\tau_s) = \left\langle \hat{E}_s^-(t+\tau_s)\hat{E}_i^-(t+\tau_i)\hat{E}_i^+(t+\tau_i)\hat{E}_s^+(t+\tau_s)\right\rangle. \quad \text{(S13)}$$

Often, since the pump beam is from a continuous wave laser, the pump spectrum is sufficiently narrow so that it can be approximately replaced by a Dirac delta function in frequency domain. One can show that the coincidence counting rate is approximately proportional to the convolution of the Fourier transforms of the filter functions for signal and idler fields. Furthermore, it depends on (i) the pump beam intensity, (ii) the efficiency of the detectors for

signal and idler fields, (iii) the magnitude of nonlinear susceptibility for SPDC, and so on. In the main text, the approximate results in Eqs. (S11) and (S12) are used to theoretically describe the quantum state, and one-photon (second-order) interferences of signal beams from triple-SPDC experimental setup are assumed to be detected. There, the idler field from the first SPDC NL crystal is allowed to interact with material of interest and various one-photon interferences (second-order correlation functions in the fields) among three signal fields are detected.

II. Cascading Configuration with Two SPDC Crystals

The experimental configuration shown in Figure S1, which corresponds to Figure 1(b) in the main text, is a schematic representation of Wang, Zou, and Mandel's experimental setup used in studying induced coherence without induced emission[S4]. It involves two second-order NL crystals for SPDC processes. The two NL crystals are pumped by the same coherent laser. Each pumped crystal can convert a single pump photon into a pair of signal and idler photons, and the dichroic mirrors (shown in deep blue in Figure S1) are used to allow signal photons pass through them and to make idler photons reflected by them. A critical importance is the optical alignment that the idler beams from the two crystals are coherently superposed to make them indistinguishable. Furthermore, the idler beams are not detected, but the one-photon interference of the associated (conjugate) signal beams, s_1 and s_2, is detected with D12 in Figure S1. To attenuate i_1, Wang *et al.*[S4] placed a 45° beam splitter (BS) between NL1 and NL2 (at the position of OS in our Figure S1), where the amplitude transmissivity and reflectivity from one side of the BS are T and R, respectively, whereas those from the other side are T' and R', respectively. They showed that the fringe visibility of one-photon interference between s_1 and s_2 detected at D12 is linearly proportional to the amplitude

transmissivity, because the degree of coherence between the conjugate idler beams is affected and reduced by the presence of the BS. Quite recently in 2014, Zeilinger and coworkers used the above cascading configuration with two down-converters (Figure S1) to experimentally demonstrate quantum imaging technique[S5], where an imaging (phase) object instead of BS was placed between NL1 and NL2. In Wang *et al.*'s experiment with a 45° BS, if the transmission coefficient T depends on idler frequency, the spectrum of T with respect to the idler frequency would correspond to an elastic scattering spectrum of the beam splitter just in the case that the BS is a lossless medium. A very interesting quantum spectroscopy experiment employing the same idea with double-SPDC scheme was performed by Kalashnikov *et al.*[S6] Since CO_2 molecules in the gas phase absorbs infrared photons, the ro-vibrational spectrum of CO_2 was obtained by measuring one-photon (second-order) interference of signal beams in *visible* frequency region. This demonstrates an IR spectroscopy with a visible photon detection instead of utilizing IR detectors.

Here, we wish to emphasize a critical difference of the experimental approach with two SPDC's from the more traditional coincidence count measurement method with a single SPDC, from the viewpoint of spectroscopic application. As shown by the Mandel group, signal and idler fields from a single SPDC are not coherent so that the corresponding second-order (in the fields) correlation function between signal and idler fields from the SPDC vanishes. Because the signal and idler fields are not coherent with each other, to investigate the signal-idler entanglement one cannot but measure coincidence counting rate or temporal correlation between signal and idler photons (Eq. (S13)). If a BS, object, or absorptive material is placed along the idler beam, the coincidence counting rate that requires detections of both signal and idler photons with two independent detectors will provide quantitative information on the property of the material, e.g., phase shift, transmission coefficient, or spatial shape (see Figure 1a). Thus, any quantum spectroscopy or imaging with just one SPDC requires detections of not

only signal photons but also idler photons. However, the double-SPDC scheme in Figure S1 (Figure 1b in the main text) is drastically different from the single-SPDC quantum spectroscopy or imaging. Regardless of any real experimental conditions with either BS, object, or absorptive material placed between NL1 and NL2, it is absolutely necessary to detect s_1 field correlation with s_2; note that s_1 is SPDC-entangled with i_1 and that the idler beam i_1 directly interacts with the material of interest. The material placed between NL1 and NL2 modulates the degree of indistinguishability or coherence between i_1 and i_2 so that the one-photon interference between the conjugate entangled s_1 and s_2 fields carries direct information on the property of material located on the idler beam pathway. There are two critical differences between the single-SPDC spectroscopy (or imaging) and the double-SPDC technique that are to be emphasized. First, the single-SPDC technique requires fourth-order (in the fields) correlation or two-photon interference measurement, whereas the double-SPDC technique needs a second-order (in the fields) correlation or one-photon interference measurement. Second, the single-SPDC technique detects not just signal photons not interacting with material but also idler photons directly interacting with material, whereas the double-SPDC technique does not require any detection of idler photons at all.

Now, unlike the low-dimensional (in terms of experimentally controlled pairs of quantum entangled photons) quantum spectroscopy (or imaging) possibilities already explored before, we shall show that a triple-SPDC technique proposed here requires detections of neither idler photons that might interact with the material of interest nor their entangled (conjugate) signal photons at all. However, still one-photon interference between two signal fields generated from the other two NL crystals involved in the second and third SPDC's in a cascading arrangement (Figure 1c in the main text) provides information on optical property of the material. Although this appears to be puzzling and quite non-intuitive, we shall show

how the critical role of vacuum field correlating idler beams makes the triple-SPDC critically differ from the double-SPDC quantum spectroscopy.

Before we present theoretical results on our triple-SPDC quantum spectroscopy, for the sake of completeness and comparison with our new results with those of double-SPDC, we here summarize the basic aspects on one-photon interference of two signal fields (s_1 and s_2) in the double-SPDC experiment shown in Figure S1 (Figure 1b in the main text). Instead of considering an imaging application, we here will focus on the case that a lossless BS is placed between NL1 and NL2 for simplicity, even though the same principle applies to quantum imaging possibility. The BS can be any four-port device with two input and two output ports. A typical BS with propagation directions at right angles or a partially reflecting film, dielectric slab, or planar material with light incident normally on both sides could be of use for BS (or OS) in Figure S1.

As emphasized earlier, the idler beam, i_1, generated by NL1 transverses through NL2 and it is perfectly aligned with the idler beam, i_2, generated by NL2. However, due to the presence of a BS between NL1 and NL2, the annihilation operator of i_2 can be related to that of i_1 as

$$\hat{a}_{i_2}(\mathbf{k}_i,\sigma_i) = [T(\mathbf{k}_i,\sigma_i)\hat{a}_{i_1}(\mathbf{k}_i,\sigma_i) + R'(\mathbf{k}_i,\sigma_i)\hat{a}_0(\mathbf{k}_i,\sigma_i)]e^{i\phi_{\text{NL1-NL2}}(\mathbf{k}_i,\sigma_i)}, \qquad \text{(S14)}$$

where $T(\mathbf{k}_i,\sigma_i)$ and $R'(\mathbf{k}_i,\sigma_i)$ are the amplitude transmissivity from the upper side of the BS (OS in Figure S1) and the reflectivity from the other side. The phase gained by the idler mode due to beam propagation from NL1 to NL2 is denoted as $\phi_{\text{NL1-NL2}}(\mathbf{k}_i,\sigma_i)$. In Eq. (S14), $\hat{a}_0$ represents the vacuum field at the unused port of the BS (OS). From now on, it is assumed that the signal and idler beams have uniform linear polarization. Then, Eq. (S14) is simplified as

$$\hat{a}_{i_2}(\mathbf{k}_i) = [T(\mathbf{k}_i)\hat{a}_{i_1}(\mathbf{k}_i) + R'(\mathbf{k}_i)\hat{a}_0(\mathbf{k}_i)]e^{i\phi_{\text{NL1-NL2}}(\mathbf{k}_i)}. \qquad \text{(S15)}$$

The transmission and reflection coefficients should satisfy the following relations: $|T(\mathbf{k}_i)|^2 + |R'(\mathbf{k}_i)|^2 = 1$, $T(\mathbf{k}_i)R'^*(\mathbf{k}_i) + T'^*(\mathbf{k}_i)R(\mathbf{k}_i) = 0$, and $T(\mathbf{k}_i)R^*(\mathbf{k}_i) + T'^*(\mathbf{k}_i)R'(\mathbf{k}_i) = 0$. The phase factor $\phi_{\text{NL1-NL2}}(\mathbf{k}_i, \sigma_i)$ in Eqs. (S14) and (S15) is mainly determined by not only the distances between the center of NL1 and the center of BS and between BS and NL2, which are denoted as $d_{\text{NL1-BS}}$ and $d_{\text{BS-NL2}}$, respectively, but also the thickness and refractive index of the BS used (Figure S2).

The phase factors gained by i_1 when it propagates from NL1 to BS and from BS to NL2 are $\phi_1(\mathbf{k}_i) = \omega_i d_{\text{NL1-BS}} / c$ and $\phi_2(\mathbf{k}_i) = \omega_i d_{\text{BS-NL2}} / c$, respectively. Thus, the phase factor $\phi_{12}(\mathbf{k}_i)$ is given as, apart from the phase delay due to the BS,

$$\phi_{\text{NL1-NL2}}(\mathbf{k}_i) = \phi_1(\mathbf{k}_i) + \phi_2(\mathbf{k}_i) = \omega_i (d_{\text{NL1-BS}} + d_{\text{BS-NL2}}) / c, \tag{S16}$$

and $\phi_{\text{NL1-NL2}}(\mathbf{k}_i)$ clearly doesn't depend on the position of the BS along the idler pathway at all. However, as we shall show later, the relative positions of beam splitters and NL crystals on the idler beam pathways are of importance in understanding various one-photon interferences in triple-SPDC experiments.

In the double-SPDC experimental configuration, even though the two pump fields for NL1 and NL2 are coherent, generations of a pair of signal and idler photons from NL1 are not correlated with those from NL2. We assume that the pump beam is a narrow band, i.e., single mode with wave vector $\mathbf{k}_p$ and frequency ω_p, uniformly polarized, and collimated at the NL crystals. Furthermore, without loss of generality, the origin of laboratory frame is at the center of NL1, i.e., $\mathbf{r}_0 = 0$. Using the SPDC Hamiltonians (Eq. (S6)) for NL1 and NL2 and the relationship between i_1 and i_2 beams given in Eq. (S15), one finds that

$$|\psi(t)\rangle = |vac\rangle + \sum_{\mathbf{k}_{s_1}}\sum_{\mathbf{k}_{i_1}} G_{s_1,i_1} A_{p_1} \mathrm{sinc}[\tfrac{(\omega_{s_1}+\omega_{i_1}-\omega_p)t}{2}] \prod_m \mathrm{sinc}[\tfrac{(\mathbf{k}_{s_1}+\mathbf{k}_{i_1}-\mathbf{k}_p)_m l_m}{2}] |\mathbf{k}_{s_1},0\rangle_{s_1,s_2} |\mathbf{k}_{i_1},0\rangle_{i_1,0}$$
$$+ \sum_{\mathbf{k}_{s_2}}\sum_{\mathbf{k}_{i_2}} G_{s_2,i_2} A_{p_2} e^{-i\phi_0} \mathrm{sinc}[\tfrac{(\omega_{s_2}+\omega_{i_2}-\omega_p)t}{2}] \prod_m \mathrm{sinc}[\tfrac{(\mathbf{k}_{s_2}+\mathbf{k}_{i_2}-\mathbf{k}_p)_m l_m}{2}]$$
$$\times \left\{ T^*(\mathbf{k}_{i_2}) |0,\mathbf{k}_{s_2}\rangle_{s_1,s_2} |\mathbf{k}_{i_2},0\rangle_{i_1,0} + R'^*(\mathbf{k}_{i_2}) |0,\mathbf{k}_{s_2}\rangle_{s_1,s_2} |0,\mathbf{k}_{i_2}\rangle_{i_1,0} \right\},$$

(S17)

where $\phi_0 = (\mathbf{k}_{s_2} + \mathbf{k}_{i_2} - \mathbf{k}_p)\cdot \mathbf{r}_{12} - i\phi_{\text{NL1-NL2}}(\mathbf{k}_{i_2})$, A_{p_j} denotes the amplitude of classical pump field in j th NL crystal, and

$$G_{s_m,i_m} = \frac{\mathrm{V}t}{2i\hbar \mathcal{V}^{3/2}} g^*_{\mathbf{k}_{s_m},\sigma_{s_m}} g^*_{\mathbf{k}_{i_m},\sigma_{i_m}} g_{\mathbf{k}_p,\sigma_p} \chi_{ijk} (e_{\mathbf{k}_{s_m},\sigma_{s_m}})^*_i (e_{\mathbf{k}_{i_m},\sigma_{i_m}})^*_j (e_{\mathbf{k}_p,\sigma_p})_k e^{i(\omega_{s_m}+\omega_{i_m}-\omega_p)t/2} \text{ (for } m=1 \text{ and } 2).$$

(S18)

In Eq. (S17) , $\mathbf{r}_{12} = \mathbf{r}_{0_2} - \mathbf{r}_{0_1}$ and the two sinc functions imply both the spatial and temporal phase-matching conditions, i.e., $\mathbf{k}_{s_m} + \mathbf{k}_{i_m} \approx \mathbf{k}_p$ and $\omega_{s_m} + \omega_{i_m} \approx \omega_p$, respectively.

Let us now consider the detection scheme in the experimental setup with two SPDC crystals in Figure S1 (Figure 1b in the main text). The two signal beams from the two crystals, NL1 and NL2, are superposed by a 50:50 beam splitter and one of the outputs of the beam splitter is detected with D12. Then, the positive frequency part of the quantized signal field at the detector D12 can be expressed as a sum of two contributions associated with s_1 and s_2, i.e.,

$$\hat{E}^{(+)}_{s,D12}(t) \propto \sum_{\mathbf{k}_{s_1}} i \exp[-i\omega_{s_1}(t-\tau_{\text{NL1-D12}})]\hat{a}_{s_1}(\mathbf{k}_{s_1}) + \sum_{\mathbf{k}_{s_2}} \exp[i\mathbf{k}_{s_2}\cdot\mathbf{r}_{12} - i\omega_{s_2}(t-\tau_{\text{NL2-D12}})]\hat{a}_{s_2}(\mathbf{k}_{s_2}),$$

(S19)

where the propagation time of s_1 from NL1 to D12 and that of s_2 from NL2 to D12 are denoted as $\tau_{\text{NL1-D12}}$ and $\tau_{\text{NL2-D12}}$, respectively, and they are related to the corresponding distances as $d_{\text{NL1-D12}}/c$ and $d_{\text{NL2-D12}}/c$. Of course, the extra phase factor due to the dispersive property of

beam splitter in front of D12 can be included in the above propagation time. The imaginary number *i* in the first term of Eq. (S19) is included because the s_1 field is reflected by the beam splitter placed before D12. If all the fields are properly normalized, $\hat{E}^{(-)}_{s,D12}\hat{E}^{(+)}_{s,D12}$ is in unit of photons per second. The average photon counting rate at D12 is given by

$$R_{s,12} = \eta \left\langle \psi(t) \,|\, \hat{E}^{(-)}_{s,D12}(t)\hat{E}^{(+)}_{s,D12}(t) \,|\, \psi(t) \right\rangle, \tag{S20}$$

where η is the quantum efficiency of the signal detector D12. Hereafter, we shall assume that the quantum efficiencies of detectors used are the same and the conversion efficiencies of all the SPDC crystals are also the same.

Inserting Eqs. (S17) and (S19) into (S20) and carrying out a straightforward calculation, one can obtain the photon counting rate at D12 that is given by, apart from a constant proportionality constant,

$$\begin{aligned} R_{s,12}(\mathbf{k}_s) \propto \left|A_{p_1}\right|^2 + \left|A_{p_2}\right|^2 + 2\left|A_{p_1}\right|\left|A_{p_2}\right|\left|T(\mathbf{k}_i)\right| \cos[\Delta\phi_{s,12}(\mathbf{k}_s) - \phi_{\text{NL1-NL2}}(\mathbf{k}_i) \\ + \phi_p - \arg\{T(\mathbf{k}_i)\} + \mathbf{k}_s \cdot \mathbf{r}_{12} + \varphi]. \end{aligned} \tag{S21}$$

where the two auxiliary phase factors $\Delta\phi_{s,12}(\mathbf{k}_s)$ and ϕ_p are defined as

$$\begin{aligned} \Delta\phi_{s,12}(\mathbf{k}_s) = \omega_s(\tau_{\text{NL2-D12}} - \tau_{\text{NL1-D12}}) = \omega_s(d_{\text{NL2-D12}} - d_{\text{NL1-D12}})/c, \\ \phi_p = \arg(A_{p_2}) - \arg(A_{p_1}) \end{aligned} \tag{S22}$$

All the remaining phase terms that are not of importance are included in φ in Eq. (S21) and ω_s denotes the center frequency of the signal beams. Eq. (S21) is the main result that was used to describe quantum imaging application by the Zeilinger group with employing the double-SPDC experimental setup in Figure S1. Note that the interference term contributing to the photon counting rate at D12 is determined by the difference in signal beam path lengths of s_1 and s_2 so that one can measure a fringe pattern by slightly modulating the position of the beam

splitter just before the D12. With the approximate result in Eq. (S21), one can show that the fringe visibility is given by

$$V_{s,12}(\mathbf{k}_s) = \frac{2\left|A_{p_1}\right|\left|A_{p_2}\right|}{\left|A_{p_1}\right|^2 + \left|A_{p_2}\right|^2}\left|T(\mathbf{k}_i)\right|. \tag{S23}$$

This simple relationship was initially obtained by Wang, Zou, and Mandel (see Eq. (19) in ref. S4). As the transmission coefficient of the beam splitter (or optical sample) placed between NL1 and NL2 increases, the indistinguishability of two signal beams at D12 increases linearly so that the fringe visibility associated with interference between signal (not idler) beams becomes large. We shall compare these results for double-SPDC experiment summarized here with those for our triple-SPDC gedankenexperiment in the following section.

III. Cascading Configuration with Three SPDC Crystals

A. Distinguishable quantum vacuum fields

The cascading-type triple-SPDC experimental setup, which is a natural expansion of the double-SPDC setup in Figure S1, is shown in Figure S3. The critical difference from the double-SPDC scheme is just to add one more SPDC crystal, NL3, to the experimental setup, which is pumped by the same coherent laser. The generated idler beam, i_3, from the NL3 is also assumed to be aligned with the other two idler beams, and the idler beam is not under detection. The signal beam s_3 is allowed to interfere with either s_1 or s_2 and the corresponding one-photon interferences are detected by D13 and D23. Since we are interested in utilizing the triple-SPDC setup for quantum spectroscopy or quantum measurement of dielectric (phase) properties of materials placed on the idler beam pathway right after the NL1, we put optical samples 1 and 2, denoted as OS1 and OS2 in Figure S3 between NL1 and NL2 and between

NL2 and NL3, respectively. We shall quantum mechanically treat them as quantum optical beam splitters. Here, it should be emphasized that the experimental configuration in Figure S3 is critically different from the triple-SPDC scheme (Figure 1c) considered in the main text. Note that, since the OS1 and OS2 in Figure S3 are right-angle (45°) beam splitters, the quantum vacuum fields, i_2' and i_0, at the unused ports of OS1 and OS2, respectively, are completely uncorrelated, i.e., distinguishable. In fact, this (Figure S3) is the experimental scheme with three SPDC crystals arranged in a cascading geometry that was considered by Ataman recently in ref. S7.

Here, it is assumed that the OS1 and OS2 are lossless materials for the sake of simplicity. The amplitude transmissivities (reflectivities) of OS1 and OS2 for incident beams propagating from left to right are $T_1(R_1)$ and $T_2(R_2)$, respectively, and those on the other sides are denoted as $T_1'(R_1')$ and $T_2'(R_2')$, respectively. We shall only focus on whether the OS1 (an object that essentially played an important role in modulating the optical coherence between s_1 and s_2 beams in double-SPDC setup in Figure S1) affects the indistinguishability of s_2 and s_3 beams generated by two other down-converters or not.

We now need to consider the following relations of idler photon annihilation operators,

$$\hat{a}_{i_2}(\mathbf{k}_i) = [T_1(\mathbf{k}_i)\hat{a}_{i_1}(\mathbf{k}_i) + R_1'(\mathbf{k}_i)\hat{a}_{i_2'}(\mathbf{k}_i)]e^{i\phi_{\text{NL1-NL2}}(\mathbf{k}_i)}, \tag{S24}$$

$$\begin{aligned}\hat{a}_{i_3}(\mathbf{k}_i) &= [T_2(\mathbf{k}_i)\hat{a}_{i_2}(\mathbf{k}_i) + R_2'(\mathbf{k}_i)\hat{a}_{i_0}(\mathbf{k}_i)]e^{i\phi_{\text{NL2-NL3}}(\mathbf{k}_i)} \\ &= [T_2(\mathbf{k}_i)T_1(\mathbf{k}_i)\hat{a}_{i_1}(\mathbf{k}_i) + T_2(\mathbf{k}_i)R_1'(\mathbf{k}_i)\hat{a}_{i_2'}(\mathbf{k}_i)]e^{i\phi_{\text{NL1-NL3}}(\mathbf{k}_i)} + R_2'(\mathbf{k}_i)\hat{a}_{i_0}(\mathbf{k}_i)e^{i\phi_{\text{NL2-NL3}}(\mathbf{k}_i)}.\end{aligned} \tag{S25}$$

Then, we can find that the quantum state becomes

$$
\begin{aligned}
|\psi(t)\rangle = |vac\rangle &+ \sum_{\mathbf{k}_{s_1}} \sum_{\mathbf{k}_{i_1}} G_{s_1,i_1} A_{p_1} \mathrm{sinc}[\tfrac{(\omega_{s_1}+\omega_{i_1}-\omega_p)t}{2}] \prod_m \mathrm{sinc}[\tfrac{(\mathbf{k}_{s_1}+\mathbf{k}_{i_1}-\mathbf{k}_p)_m l_m}{2}] |\mathbf{k}_{s_1},0,0\rangle_{s_1,s_2,s_3} |\mathbf{k}_{i_1},0,0\rangle_{i_1,i_2',i_0} \\
&+ \sum_{\mathbf{k}_{s_2}} \sum_{\mathbf{k}_{i_2}} G_{s_2,i_2} A_{p_2} e^{-i\phi_0} \mathrm{sinc}[\tfrac{(\omega_{s_2}+\omega_{i_2}-\omega_p)t}{2}] \prod_m \mathrm{sinc}[\tfrac{(\mathbf{k}_{s_2}+\mathbf{k}_{i_2}-\mathbf{k}_p)_m l_m}{2}] \\
&\times \left\{ T_1^*(\mathbf{k}_{i_2}) |0,\mathbf{k}_{s_2},0\rangle_{s_1,s_2,s_3} |\mathbf{k}_{i_2},0,0\rangle_{i_1,i_2',i_0} + R_1'^*(\mathbf{k}_{i_2}) |0,\mathbf{k}_{s_2},0\rangle_{s_1,s_2,s_3} |0,\mathbf{k}_{i_2},0\rangle_{i_1,i_2',i_0} \right\} \\
&+ \sum_{\mathbf{k}_{s_3}} \sum_{\mathbf{k}_{i_3}} G_{s_3,i_3} A_{p_3} e^{-i\phi_1} \mathrm{sinc}[\tfrac{(\omega_{s_3}+\omega_{i_3}-\omega_p)t}{2}] \prod_m \mathrm{sinc}[\tfrac{(\mathbf{k}_{s_3}+\mathbf{k}_{i_3}-\mathbf{k}_p)_m l_m}{2}] \\
&\times \{ T_2^*(\mathbf{k}_{i_3}) T_1^*(\mathbf{k}_{i_3}) |0,0,\mathbf{k}_{s_3}\rangle_{s_1,s_2,s_3} |\mathbf{k}_{i_3},0,0\rangle_{i_1,i_2',i_0} + T_2^*(\mathbf{k}_{i_3}) R_1'^*(\mathbf{k}_{i_3}) |0,0,\mathbf{k}_{s_3}\rangle_{s_1,s_2,s_3} |0,\mathbf{k}_{i_3},0\rangle_{i_1,i_2',i_0} \\
&\quad + R_2'^*(\mathbf{k}_{i_3}) |0,0,\mathbf{k}_{s_3}\rangle_{s_1,s_2,s_3} e^{\phi_{\text{NL1-NL2}}(\mathbf{k}_{i_3})} |0,0,\mathbf{k}_{i_3}\rangle_{i_1,i_2',i_0} \}
\end{aligned}
\tag{S26}
$$

where $\phi_1 = (\mathbf{k}_{s_3} + \mathbf{k}_{i_3} - \mathbf{k}_p) \cdot \mathbf{r}_{13} - i\phi_{\text{NL1-NL3}}(\mathbf{k}_{i_3})$ and A_{p_j} denotes an amplitude of classical pump field in the j th NL crystal. If pump intensities at the three crystals are assumed to be the same and the phase matching conditions are assumed to be satisfied, the visibilities at D12, D13 and D23 are found to be

$$
V_{s,12}(\mathbf{k}_s) = \frac{2|A_{p_1}||A_{p_2}|}{|A_{p_1}|^2 + |A_{p_2}|^2} |T_1(\mathbf{k}_i)|, \tag{S27}
$$

$$
V_{s,13}(\mathbf{k}_s) = \frac{2|A_{p_1}||A_{p_3}|}{|A_{p_1}|^2 + |A_{p_3}|^2} |T_1(\mathbf{k}_i) T_2(\mathbf{k}_i)|, \tag{S28}
$$

$$
V_{s,23}(\mathbf{k}_s) = \frac{2|A_{p_2}||A_{p_3}|}{|A_{p_2}|^2 + |A_{p_3}|^2} |T_2(\mathbf{k}_i)|. \tag{S29}
$$

We can easily see that the fringe visibility of the one-photon (second-order) interference between s_2 and s_3 beams, $V_{s,23}$ in Eq. (S29), does not depend on the optical property of OS1, T_1, as expected.

These results can be easily understood by considering the reduced density matrix of signal beams given by $\rho^s = \mathrm{Tr}_i \rho^t$. The amplitude of coherence term of s_2 and s_3, which is the

off diagonal element of the corresponding density matrix, ρ_{23}, can be written as the sum of $T_2 |T_1|^2$ and $T_2 |R_1'|^2$. We note that $T_2 |T_1|^2$ is essentially the degree of induced coherence by $\left|\mathbf{k}_{i_1}\right\rangle = \left|\mathbf{k}_{i_2}\right\rangle_{i_2} = \left|\mathbf{k}_{i_3}\right\rangle_{i_3}$, and $T_2 |R_1'|^2$ is that by $\left|\mathbf{k}_{i_2'}\right\rangle_{i_2'} = \left|\mathbf{k}_{i_2}\right\rangle_{i_2} = \left|\mathbf{k}_{i_3}\right\rangle_{i_3}$. Therefore, the degree of coherence between s_2 and s_3 beams naturally becomes independent of T_1 due to the following relationship $|T_1|^2 + |R_1'|^2 = 1$. In conclusion, if the two beam splitters (optical samples OS1 and OS2) placed in a triple-SPDC setup, where the three SPDC crystals are aligned in a cascading geometry, are 45° (or more generally non-orthogonal) to the idler beam propagation direction, due to the complete distinguishability between the two quantum vacuum fields at the two unused ports of the beam splitters one cannot extract any spectral (or phase) information of the material of interest, OS1, through any one-photon (second-order) interference measurement of two signal beams, s_1 and s_2, with D23.

B. Indistinguishable quantum vacuum fields: Optical resonator configuration

Now, we consider that the optical sample cell surfaces of OS1 and OS2 are normal to the incident idler beam (Figure S4). In the case of double-SPDC with a single BS (or OS) in Figure S1 (Figure 1b in the main text), the BS could be either a usual BS with beam propagation directions at right angles or a partially reflecting film or material with light incident normally on both sides. The results for the double-SPDC experiment do not depend on the angle of BS with respect to idler beam propagation direction at all. However, in the present case of the triple-SPDC experiment considered here (Figure S4 or Figure 1c in the main text), the OS1 and OS2 are partially reflecting planar materials (or dielectric slabs) of which surfaces are normal to incident beams. This seemingly minor difference between the two configurations shown in Figures S3 and S4 has been found to be extremely important as shown below.

The quantum mechanical description of such partially reflecting film is still identical to that of conventional BS. The pair of OS1 and OS2 are therefore capable of forming a planar mirror resonator when they have non-zero reflection coefficients, which differs from the optical setup considered by Ataman recently[S7], where he examined a special quantum imaging possibility of a similar but critically different triple-SPDC setup. He considered two right-angle (45°) beam splitters at the positions of OS1 and OS2 so that there are two uncorrelated vacuum (see Figure S3). Furthermore, the detection scheme considered in ref. S7 is completely different from ours depicted in Figure S4 (Figure 1c in the main text).

Now, due to the existence of a resonator formed by OS1 and OS2 in Figure S4, the common vacuum mode is subject to the same planar materials, OS1 and OS2. Again, we shall particularly consider how the OS1 affects the indistinguishability of s_2 and s_3 beams. Note that neither s_1 nor i_1 beams that are generated by the so-called radiation source, NL1 crystal, arrives at the detector D23 at all, but the induced coherence among idler beams by means of aligning them in a collinear configuration and the indistinguishability of two quantum vacuum fields at the unused ports of OS1 and OS2 are shown to be critical in measuring optical properties of material (OS1) of interest placed after the spectroscopic radiation source NL1. In fact, this is quite counter-intuitive because neither the i_1 beams that could directly interact with material nor the s_1 beams that are correlated with i_1 beams via SPDC at NL1 are under the one-photon interference measurements with D23 at all.

Before we present the theoretical expression on the quantum state after all three SPDC processes, it would be necessary to discuss about the quantum description of idler state when all three idler beams are aligned like Figure S4. In Figure S5, the key components and definitions of distances are depicted. To obtain the idler field amplitudes at NL2 and NL3 that

are related to the idler field i_1 generated by NL1 crystal, let us first consider the amplitude of idler field i_2 at the position of NL2 that is given by

$$\hat{a}_{i_2} = T_1\hat{a}_{i_1}\exp[i(\phi_{\text{NL1-NL2}})] + (R_1'R_2\hat{a}_{i_2} + R_1'T_2'\hat{a}_0)\exp(i\kappa) \tag{S30}$$

where the phase factors are defined as $\kappa = 2\omega_i(d_{\text{NL1-OS1}} + d_{\text{OS1-NL2}})/c$. The phase factor κ is that gained by the idler beam when it undergoes a single cycle of the resonator formed by OS1 and OS2. Due to the vacuum field on the right-hand side of OS2 in Figure S5, there appears the third term on the right-hand side of Eq. (S30). Then, solving Eq. (S30) for the amplitude of i_2, one can find that the relation of $\hat{a}_{i_2}$ with $\hat{a}_{i_1}$ and $\hat{a}_0$, i.e.,

$$\hat{a}_{i_2} = \frac{T_1 e^{i\phi_{\text{NL1-NL2}}}}{1 - R_1'R_2 e^{i\kappa}}\hat{a}_{i_1} + \frac{R_1'T_2'e^{i\kappa}}{1 - R_1'R_2 e^{i\kappa}}\hat{a}_0. \tag{S31}$$

Here, the term $R_1'R_2e^{i\kappa}$ in the denominator can be identified as the complex round-trip amplitude attenuation factor of idler photons inside the resonator.

Now, the amplitude of the idler beam i_3 at the position of NL3 is

$$\begin{aligned} \hat{a}_{i_3} &= T_2 e^{i\phi_{\text{NL2-NL3}}}\hat{a}_{i_2} + R_2' e^{i\phi_{\text{NL2-NL3}}}\hat{a}_0 \\ &= \frac{T_1T_2 e^{i\phi_{\text{NL1-NL2}} + i\phi_{\text{NL2-NL3}}}}{1 - R_1'R_2 e^{i\kappa}}\hat{a}_{i_1} + \left\{\frac{R_2' + R_1'(T_2T_2' - R_2R_2')e^{i\kappa}}{1 - R_1'R_2 e^{i\kappa}}\right\} e^{i\phi_{\text{NL2-NL3}}}\hat{a}_0. \end{aligned} \tag{S32}$$

If the OS2 is a lossless beam splitter satisfying energy balance condition, the term, $T_2T_2' - R_2R_2'$, on the right-hand side of Eq. (S32) can be further simplified as $T_2T_2' - R_2R_2' = T_2'/T_2^* = -R_2'/R_2^*$. Using these results for the annihilation operators of the three idler photons and the SPDC Hamiltonians describing the three SPDC processes, we obtain the quantum state of light after triple SPDC's that is

$$
\begin{aligned}
|\psi(t)\rangle = |vac\rangle &+ \sum_{\mathbf{k}_{s_1}}\sum_{\mathbf{k}_{i_1}} G_{s_1,i_1} A_{p_1} \sin c[\tfrac{(\omega_{s_1}+\omega_{i_1}-\omega_p)t}{2}] \prod_m \text{sinc}[\tfrac{(\mathbf{k}_{s_1}+\mathbf{k}_{i_1}-\mathbf{k}_p)_m l_m}{2}] |\mathbf{k}_{s_1},0,0\rangle_{s_1,s_2,s_3} |k_{i_1},0\rangle_{i_1,i_0} \\
&+ \sum_{\mathbf{k}_{s_2}}\sum_{\mathbf{k}_{i_2}} G_{s_2,i_2} A_{p_2} e^{-i(\mathbf{k}_{s_2}+\mathbf{k}_{i_2}-\mathbf{k}_p)\cdot\mathbf{r}_{12}} \text{sinc}[\tfrac{(\omega_{s_2}+\omega_{i_2}-\omega_p)t}{2}] \prod_m \text{sinc}[\tfrac{(\mathbf{k}_{s_2}+\mathbf{k}_{i_2}-\mathbf{k}_p)_m l_m}{2}] \\
&\times \frac{1}{\sqrt{\mathcal{N}}} \left\{ [\frac{T_1(\mathbf{k}_{i_2}) e^{i\phi_{\text{NL1-NL2}}}}{1-R_1'(\mathbf{k}_{i_2}) R_2(\mathbf{k}_{i_2}) e^{i\kappa}}]^* |0,\mathbf{k}_{s_2},0\rangle_{s_1,s_2,s_3} |\mathbf{k}_{i_2},0\rangle_{i_1,i_0} + [\frac{R_1'(\mathbf{k}_{i_2}) T_2'(\mathbf{k}_{i_2}) e^{i\kappa}}{1-R_1'(\mathbf{k}_{i_2}) R_2(\mathbf{k}_{i_2}) e^{i\kappa}}]^* |0,\mathbf{k}_{s_2},0\rangle_{s_1,s_2,s_3} |0,\mathbf{k}_{i_2}\rangle_{i_1,i_0} \right\} \\
&+ \sum_{\mathbf{k}_{s_3}}\sum_{\mathbf{k}_{i_3}} G_{s_3,i_3} A_{p_3} e^{-i(\mathbf{k}_{s_3}+\mathbf{k}_{i_3}-\mathbf{k}_p)\cdot\mathbf{r}_{13}} \text{sinc}[\tfrac{(\omega_{s_3}+\omega_{i_3}-\omega_p)t}{2}] \prod_m \text{sinc}[\tfrac{(\mathbf{k}_{s_3}+\mathbf{k}_{i_3}-\mathbf{k}_p)_m l_m}{2}] \\
&\times \{ [\frac{T_1(\mathbf{k}_{i_3}) T_2(\mathbf{k}_{i_3}) e^{i\phi_{\text{NL1-NL2}}+i\phi_{\text{NL2-NL3}}}}{1-R_1'(\mathbf{k}_{i_3}) R_2(\mathbf{k}_{i_3}) e^{i\kappa}}]^* |0,0,\mathbf{k}_{s_3}\rangle_{s_1,s_2,s_3} |\mathbf{k}_{i_3},0\rangle_{i_1,i_0} \\
&+ [(\frac{R_2'(\mathbf{k}_{i_3}) + R_1'(\mathbf{k}_{i_3})(T_2(\mathbf{k}_{i_3}) T_2'(\mathbf{k}_{i_3}) - R_2(\mathbf{k}_{i_3}) R_2'(\mathbf{k}_{i_3})) e^{i\kappa}}{1-R_1'(\mathbf{k}_{i_3}) R_2(\mathbf{k}_{i_3}) e^{i\kappa}}) e^{i\phi_{\text{NL2-NL3}}}]^* (\mathbf{k}_{i_3}) |0,0,\mathbf{k}_{s_3}\rangle_{s_1,s_2,s_3} |0,\mathbf{k}_{i_3}\rangle_{i_1,i_0} \}
\end{aligned}
$$

(S33)

where $\mathcal{N}$ is a normalization factor, $\mathcal{N} = |\frac{T_1(\mathbf{k}_{i_2}) e^{i\phi_{\text{NL1-NL2}}}}{1-R_1'(\mathbf{k}_{i_2}) R_2(\mathbf{k}_{i_2}) e^{i\kappa}}|^2 + |\frac{R_1'(\mathbf{k}_{i_2}) T_2'(\mathbf{k}_{i_2}) e^{i\kappa}}{1-R_1'(\mathbf{k}_{i_2}) R_2(\mathbf{k}_{i_2}) e^{i\kappa}}|^2$. We note the normalization is required during the transformation from idler 2 (i_2) to idler 1 (i_1) and vacuum (i_0).

We next consider the detection scheme in the experimental setup with three SPDC crystals in Figure S4. A pair of signal fields s_j and s_k from NL_j and NL_k are superposed by a properly placed beam splitter and its one-photon interference is detected by D*jk*, as shown in Figure S4. Depending on detailed positions and beam paths in the experimental setup, the signal beams could be attenuated and/or gain extra phase terms. Apart from the constant factors, the positive frequency parts of the quantized signal fields at the detectors can be expressed as

$$\hat{E}_{s,D12}^{(+)}(t) \propto \sum_{\mathbf{k}_{s_1}} e^{-i\omega_{s_1}t+i\phi_{\text{NL1-D12}}} \hat{a}_{s_1}(\mathbf{k}_{s_1}) + \sum_{\mathbf{k}_{s_2}} e^{i\mathbf{k}_{s_2}\cdot\mathbf{r}_{12}-i\omega_{s_2}t+i\phi_{\text{NL2-D12}}} \hat{a}_{s_2}(\mathbf{k}_{s_2})$$
$$\hat{E}_{s,D13}^{(+)}(t) \propto \sum_{\mathbf{k}_{s_1}} e^{-i\omega_{s_1}t+i\phi_{\text{NL1-D13}}} \hat{a}_{s_1}(\mathbf{k}_{s_1}) + \sum_{\mathbf{k}_{s_3}} e^{i\mathbf{k}_{s_3}\cdot\mathbf{r}_{13}-i\omega_{s_3}t+i\phi_{\text{NL3-D13}}} \hat{a}_{s_3}(\mathbf{k}_{s_3}) \tag{S34}$$
$$\hat{E}_{s,D23}^{(+)}(t) \propto \sum_{\mathbf{k}_{s_2}} e^{i\mathbf{k}_{s_2}\cdot\mathbf{r}_{12}-i\omega_{s_2}t+i\phi_{\text{NL2-D23}}} \hat{a}_{s_2}(\mathbf{k}_{s_2}) + \sum_{\mathbf{k}_{s_3}} e^{i\mathbf{k}_{s_3}\cdot\mathbf{r}_{13}-i\omega_{s_3}t+i\phi_{\text{NL3-D23}}} \hat{a}_{s_3}(\mathbf{k}_{s_3})$$

where the phase term, for instance $\phi_{\text{NL1-D12}}$, is largely determined by the distance $d_{\text{NL1-D12}}$ from NL1 to D12, i.e., $\phi_{\text{NL1-D12}} = \omega_{s_1}\tau_{\text{NL1-D12}} = \omega_{s_1} d_{\text{NL1-D12}} / c$ as well as the phase gained by the corresponding signal field due to the presence of beam splitters placed before D12.

Now, the three one-photon interferences are detected by measuring the average photon counting rates at detectors that are given by

$$R_{s,12} = \eta \left\langle \psi(t) \,|\, \hat{E}_{s,D12}^{(-)}(t)\hat{E}_{s,D12}^{(+)}(t) \,|\, \psi(t) \right\rangle$$
$$R_{s,13} = \eta \left\langle \psi(t) \,|\, \hat{E}_{s,D13}^{(-)}(t)\hat{E}_{s,D13}^{(+)}(t) \,|\, \psi(t) \right\rangle, \tag{S35}$$
$$R_{s,23} = \eta \left\langle \psi(t) \,|\, \hat{E}_{s,D23}^{(-)}(t)\hat{E}_{s,D23}^{(+)}(t) \,|\, \psi(t) \right\rangle$$

where we assume that the quantum efficiency η of the three detectors are the same. Following the same arguments and carrying out a long but relatively straightforward calculation, we could obtain the photon counting rates at the detectors.

First of all, let us consider the photon counting rate at D12, which is found to be, apart from a constant proportionality constant,

$$R_{s,12} \propto |A_{p_1}|^2 + |A_{p_2}|^2 + 2|A_{p_1}||A_{p_2}| \frac{1}{\sqrt{\mathcal{N}}} \left| \frac{T_1(\mathbf{k}_{i_2}) e^{i\phi_{\text{NL1-NL2}}}}{1 - R_1'(\mathbf{k}_{i_2}) R_2(\mathbf{k}_{i_2}) e^{i\kappa}} \right|$$
$$\times \cos\left[\Delta\phi_{s,12} + \phi_{p_{12}} - \arg\left\{ \frac{T_1(\mathbf{k}_{i_2}) e^{i\phi_{\text{NL1-NL2}}}}{1 - R_1'(\mathbf{k}_{i_2}) R_2(\mathbf{k}_{i_2}) e^{i\kappa}} \right\} + \mathrm{k}_{s_2} \cdot \mathrm{r}_{12} + \varphi_{12} \right] \tag{S36}$$

where the two auxiliary phase factors $\Delta\phi_{s,12}(\mathbf{k}_s)$ and $\phi_{p_{12}}$ are defined as

$$\Delta\phi_{s,12} = \phi_{\text{NL2-D12}}(\mathbf{k}_{s_2}) - \phi_{\text{NL1-D12}}(\mathbf{k}_{s_1}),$$
$$\phi_{p_{12}} = \arg(A_{p_2}) - \arg(A_{p_1}). \tag{S37}$$

All the remaining phase terms that are not of importance are included in φ_{12} in Eq. (S36). Since the photon counting rate at D12 depends on the difference in signal beam path lengths of s_1 and s_2, one should be able to observe a fringe pattern by slightly modulating the position of a BS just before the D12. Then, the fringe visibility at D12 is found to be

$$V_{s,12} = \frac{2|A_{p_1}||A_{p_2}|\left|\dfrac{T_1(\mathbf{k}_{i_2})e^{i\phi_{\text{NL1-NL2}}}}{1-R_1'(\mathbf{k}_{i_2})R_2(\mathbf{k}_{i_2})e^{i\kappa}}\right|}{(|A_{p_1}|^2+|A_{p_2}|^2)\sqrt{\mathcal{N}}} \tag{S38}$$

and plotted in Figure S6a. The visibility shows a complicated dependence on the transmission and reflection coefficients of OS1 and OS2. To confirm that the above result is consistent with the previous findings with simpler experimental configuration with two SPDC crystals, let us consider a limiting case that the optical sample 2, OS2, placed between NL2 and NL3 is removed. In this limiting case, for OS2, the reflection coefficient is zero and the transmission coefficient is unity. Then, Eq. (S38) becomes identical to Eq. (S23) obtained for the double-SPDC experiment, which confirms the validity of the theoretical result in Eq. (S38).

Next, let us consider the one-photon interference between s_1 and s_3. The photon counting rate at D13 is found to be

$$\begin{aligned} R_{s,13} \propto\ & |A_{p_1}|^2 + |A_{p_3}|^2 + 2|A_{p_1}||A_{p_3}|\left|\frac{T_1(\mathbf{k}_{i_3})T_2(\mathbf{k}_{i_3})}{1-R_1'(\mathbf{k}_{i_3})R_2(\mathbf{k}_{i_3})e^{i\kappa(\mathbf{k}_{i_3})}}\right| \\ & \times\cos\left[\Delta\phi_{s,13}+\phi_{p_{13}}-\arg\left\{\frac{T_1(\mathbf{k}_{i_3})T_2(\mathbf{k}_{i_3})e^{i\phi_{\text{NL1-NL2}}(\mathbf{k}_{i_3})+i\phi_{\text{NL2-NL3}}(\mathbf{k}_{i_3})}}{1-R_1'(\mathbf{k}_{i_3})R_2(\mathbf{k}_{i_3})e^{i\kappa}}\right\}+\mathbf{k}_s\cdot\mathbf{r}_{13}+\varphi_{13}\right], \end{aligned} \tag{S39}$$

where

$$\begin{aligned} \Delta\phi_{s,13} &= \phi_{\text{NL3-D13}}(\mathbf{k}_{s_3}) - \phi_{\text{NL1-D13}}(\mathbf{k}_{s_1}), \\ \phi_{p_{13}} &= \arg(A_{p_3}) - \arg(A_{p_1}) \end{aligned} \tag{S40}$$

φ_{13} contains the remaining phase terms. The photon counting rate at D13 depends on the difference in path lengths of s_1 and s_3, which is manifested by $\Delta\phi_{s,13}$, so that it would exhibit a fringe pattern. The corresponding visibility is then given by

$$V_{s,13} = \frac{2|A_{p_1}||A_{p_3}|\left|\dfrac{T_1(\mathbf{k}_{i_3})T_2(\mathbf{k}_{i_3})e^{i\phi_{\text{NL1-NL2}}(\mathbf{k}_{i_3})+i\phi_{\text{NL2-NL3}}(\mathbf{k}_{i_3})}}{1-R_1^{'}(\mathbf{k}_{i_3})R_2(\mathbf{k}_{i_3})e^{i\kappa}}\right|}{|A_{p_1}|^2+|A_{p_3}|^2}. \quad \text{(S41)}$$

If there is no OS2 on the idler beam pathway, i.e., $T_2 = T_2^{'} = 1$ and $R_2 = R_2^{'} = 0$, the photon counting rate at D13 in Eq. (S41) is simplified as

$$V_{s,13} = \frac{2|A_{p_1}||A_{p_3}|}{|A_{p_1}|^2+|A_{p_3}|^2}|T_1(\mathbf{k}_{i_3})| \quad \text{(S42)}$$

As plotted in Figure S7, the visibility depends on the overlap between i_1 and i_3 states that correspond to the input field and output field of the resonator. If the pump intensity at NL3 is the same with that at NL2, the above expression (Eq. (S42)) for $V_{s,13}$ equals $V_{s,12}$ in Eq. (S38), as expected. Again, the reduced expression for the special case when no OS2 is used becomes identical to Eq. (S23), as expected. However, the general result in Eq. (S41) is more involved and shows that the degree of indistinguishability of s_1 from s_3 depends on the optical properties of OS2 in a complicated but calculable way.

The more interesting one-photon interference phenomenon is how the optical properties of OS1 (target sample) modulate the photon counting rate at D23. Note that the signals s_2 and s_3 are generated by the NL2 and NL3 that are placed after the NL1, where NL1 is considered to be the radiation source generating s_1 and i_1 photons under consideration for spectroscopic (imaging) application here. Note that the beam that directly interacts with our target sample, OS1, is i_1 not i_2 nor i_3, whereas the photons detected by D23 are s_2 and s_3 beams that neither

interacted with OS1 nor were produced by the same radiation source NL1 via the corresponding SPDC process at NL1. Using Eqs. (S33)-(S35), we find that the photon counting rate at D23 is given by

$$
\begin{aligned}
R_{s,23} \propto & \left|A_{p_2}\right|^2+\left|A_{p_3}\right|^2+\frac{2\left|A_{p_2}\right|\left|A_{p_3}\right|}{\sqrt{\mathcal{N}}}\left|\frac{T_1(\mathbf{k}_{i_2})T_1^*(\mathbf{k}_{i_3})T_2^*(\mathbf{k}_{i_3})}{\left(1-R_1'(\mathbf{k}_{i_2})R_2(\mathbf{k}_{i_2})e^{i\kappa(\mathbf{k}_{i_2})}\right)\left(1-R_1'(\mathbf{k}_{i_3})R_2(\mathbf{k}_{i_3})e^{i\kappa(\mathbf{k}_{i_3})}\right)^*}\right| \\
& \times\cos\left[\Delta\phi_{s,23}(\mathbf{k}_s)+\phi_{p_{23}}-\arg\left\{\frac{T_1^*(\mathbf{k}_{i_2})T_1(\mathbf{k}_{i_3})T_2(\mathbf{k}_{i_3})e^{i\phi_{\text{NL2-NL3}}(\mathbf{k}_{i_3})}}{\left(1-R_1'(\mathbf{k}_{i_2})R_2(\mathbf{k}_{i_2})e^{i\kappa(\mathbf{k}_{i_2})}\right)^*\left(1-R_1'(\mathbf{k}_{i_3})R_2(\mathbf{k}_{i_3})e^{i\kappa(\mathbf{k}_{i_3})}\right)}\right\}+\mathbf{k}_{s_3}\cdot\mathbf{r}_{13}-\mathbf{k}_{s_2}\cdot\mathbf{r}_{12}+\varphi_{23}\right] \\
& +\frac{2\left|A_{p_2}\right|\left|A_{p_3}\right|}{\sqrt{\mathcal{N}}}\left|\frac{R_1'(\mathbf{k}_{i_2})T_2'(\mathbf{k}_{i_2})[R_2'(\mathbf{k}_{i_3})+R_1'(\mathbf{k}_{i_3})[T_2(\mathbf{k}_{i_3})T_2'(\mathbf{k}_{i_3})-R_2(\mathbf{k}_{i_3})R_2'(\mathbf{k}_{i_3})]e^{i\kappa(\mathbf{k}_{i_3})}]^*}{\left(1-R_1'(\mathbf{k}_{i_2})R_2(\mathbf{k}_{i_2})e^{i\kappa(\mathbf{k}_{i_2})}\right)\left(1-R_1'(\mathbf{k}_{i_3})R_2(\mathbf{k}_{i_3})e^{i\kappa(\mathbf{k}_{i_3})}\right)^*}\right| \\
& \times\cos\Big[\Delta\phi_{s,23}(\mathbf{k}_s)+\phi_{p_{23}}+\mathbf{k}_{s_3}\cdot\mathbf{r}_{13}-\mathbf{k}_{s_2}\cdot\mathbf{r}_{12}+\varphi_{23} \\
& -\arg\left\{\frac{[R_1'^*(\mathbf{k}_{i_2})T_2'^*(\mathbf{k}_{i_2})[R_2'(\mathbf{k}_{i_3})+R_1'(\mathbf{k}_{i_3})[T_2(\mathbf{k}_{i_3})T_2'(\mathbf{k}_{i_3})-R_2(\mathbf{k}_{i_3})R_2'(\mathbf{k}_{i_3})]e^{i\kappa(\mathbf{k}_{i_3})}]]e^{i\phi_{\text{NL2-NL3}}(\mathbf{k}_{i_3})-i\kappa(\mathbf{k}_{i_2})}}{\left(1-R_1'(\mathbf{k}_{i_2})R_2(\mathbf{k}_{i_2})e^{i\kappa(\mathbf{k}_{i_2})}\right)^*\left(1-R_1'(\mathbf{k}_{i_3})R_2(\mathbf{k}_{i_3})e^{i\kappa(\mathbf{k}_{i_3})}\right)}\right\}\Bigg],
\end{aligned}
\tag{S43}
$$

where

$$
\begin{aligned}
\Delta\phi_{s,23} &= \phi_{\text{NL3-D23}}(\mathbf{k}_{s_3})-\phi_{\text{NL2-D23}}(\mathbf{k}_{s_2}), \\
\phi_{p_{13}} &= \arg(A_{p_3})-\arg(A_{p_1}).
\end{aligned}
\tag{S44}
$$

Again, φ_{23} contains extra phase terms. The photon counting rate at D23 depends on the difference in path lengths of s_2 and s_3 so that one can expect a fringe with finite visibility. For the sake of simplicity, we shall assume that the wave vector and frequency of idler 2 are the same with those of idler 3, which is realizable by accurately aligning the idler beams. With this assumption, we could obtain the fringe visibility, $V_{s,23}$, that is given as

$$
V_{s,23}=\frac{2\left|A_{p_2}\right|\left|A_{p_3}\right|}{\left|A_{p_2}\right|^2+\left|A_{p_3}\right|^2}\,\frac{\left||T_1|^2\,T_2+R_1'^*T_2'^*(R_2'+R_1'[T_2T_2'-R_2R_2']e^{i\kappa})e^{-i\kappa}\right|}{\sqrt{\mathcal{N}}\left|1-R_1'R_2e^{i\kappa}\right|^2},
\tag{S45}
$$

where $\mathcal{N} = (|T_1|^2 + |R_1' T_2|^2)/|1 - R_1' R_2 e^{i\kappa}|^2$. If we assume that OS1 and OS2 are symmetric dielectric slabs with $T_j = T_j'$ and $R_j = R_j' = i\sqrt{1-T_j^2}$ (for $j = 1, 2$), the fringe visibility at D23 is simplified as

$$V_{s,23} = \frac{2|A_{p_2}||A_{p_3}|}{|A_{p_2}|^2 + |A_{p_3}|^2} \frac{|T_2|}{\sqrt{\mathcal{N}}\,|1 - R_1' R_2 e^{i\kappa}|} = \frac{2|A_{p_2}||A_{p_3}|}{|A_{p_2}|^2 + |A_{p_3}|^2} \frac{|T_2|}{\sqrt{|T_1|^2 + |T_2|^2 - |T_1|^2 |T_2|^2}}. \quad \text{(S46)}$$

Interestingly, $V_{s,23}$ is independent of κ, which is the phase gained by an idler field in the optical resonator. To obtain Eq. (S46), one should take into account the difference in the argument terms from two interference terms in Eq. (S43). From Eq. (S45), when there is no OS2 along the idler pathway, i.e., $T_2 = T_2' = 1$ and $R_2 = R_2' = 0$, the visibility $V_{s,23}$ reduces to

$$V_{s,23} = \frac{2|A_{p_1}||A_{p_3}|}{|A_{p_1}|^2 + |A_{p_3}|^2}. \quad \text{(S47)}$$

This result in the case of no OS2 indicates that, regardless of the presence of optical sample OS1 placed between NL1 and NL2, the s_2 and s_3 beams are always maximally indistinguishable and their one-photon interference approaches its maximum and furthermore does not depend on the properties of OS1 at all. Nonetheless, this is not of main interest because our goal is to show that the optical properties of OS1 can be studied by means of detecting one-photon interference between s_2 and s_3 photons. As a matter of fact, the more general result on the visibility $V_{s,23}$ in Eq. (S45) and in Figure S6b indeed shows that the visibility $V_{s,23}$ does depend on the transmission (and reflection) coefficient of OS1.

In the main text, we present discussions on a few limiting cases of the general results given here. It is also shown that, controlling the positions of OS2 relative to OS1, one can indirectly measure the reflection coefficient of OS1 by analyzing one-photon interference of s_2 and s_3. Furthermore, we show that the quantum nature of the three quantum entangled signal

states generated by three SPDC processes coherently ‘induced’ by aligning three idler fields as well as vacuum fields can be described in terms of a general multipartite quantum entanglement.

Supplementary References

S1. C. K. Hong and L. Mandel, *Phys. Rev. A* **31**, 2409 (1985).

S2. S. P. Walborn, C. H. Monken, S. Padua, and P. H. Souto Ribeiro, *Phys. Rep.* **495**, 87 (2010).

S3. M. Lahiri, R. Lapkiewicz, G. B. Lemos, and A. Zeilinger, *Phys. Rev. A* **92**, 013832 (2015).

S4. X. Y. Zou, L. J. Wang and L. Mandel, *Phys. Rev. Lett.* **67**, 318 (1991) ; L. J. Wang, X. Y. Zou, and L. Mandel, *Phys. Rev. A* **44**, 4614 (1991).

S5. G. B. Lemos, V. Borish, G. D. Cole, S. Ramelow, R. Lapkiewicz, and A. Zeilinger, *Nature* **512**, 409 (2014)

S6. D. A. Kalashnikov, E. V. Melik-Gaykazyan, A. A. Kalachev, Y. F. Yu, A. I. Kuznetsov and L. A. Krivitskyi, *arXiv:1611.02415* (2016).

S7. S. Ataman, *Eur. Phys. J. D* **70**, 127 (2016).

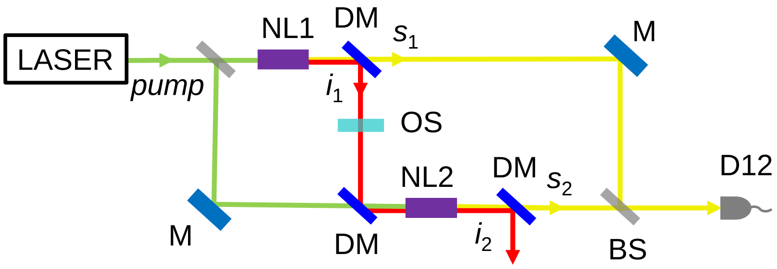

Figure S1. A schematic layout of quantum spectroscopy or imaging experiment with two SPDC crystals. Two nonlinear (NL) crystals, NL1 and NL2, are pumped by a common coherent laser. The generated signal and idler photons are separated by a dichroic mirror (DM). Idler beam, i_1, from NL1 is allowed to interact with optical sample (OS) and is aligned collinearly with the idler 2, i_2, from NL2. The idler beams are not under detection, but the one-photon interference, second order interference, between two signal beams, s_1 and s_2, is measured with detector D12.

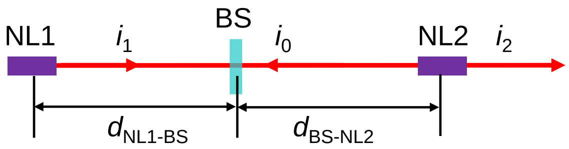

Figure S2. The distances between NL1 and BS (or OS in Figure S1) and between BS (OS) and NL2.

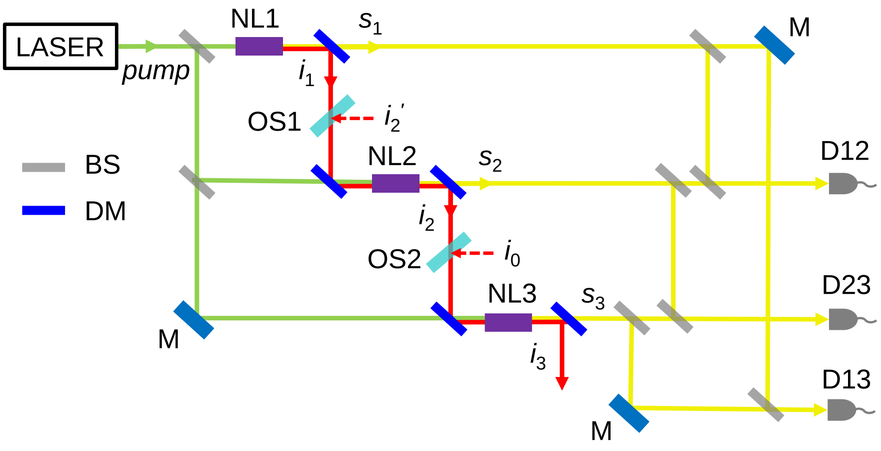

Figure S3. A schematic layout of quantum spectroscopy or imaging experiment with three SPDC crystals in a cascading arrangement. Three nonlinear (NL) crystals, NL1, NL2, and NL2, are pumped by the same coherent laser. The generated signal and idler photons are separated by a dichroic mirror (DM). Idler beam, i_1, from NL1 is allowed to interact with 45° tilted Beam Splitter 1 (or Optical Sample 1, OS1) and is aligned collinearly with the idler 2, i_2, from NL2. Similarly, the idler 2, i_2, is allowed to interact with 45° tilted Beam Splitter 2 (OS2 in this figure) and is perfectly aligned in a way that the idler 3 cannot be distinguished from the other idler beams. The two quantum vacuum modes at the unused ports of OS1 and OS2 are denoted as i_2' and i_0, respectively. The idler beams are not detected, but the one-photon interferences, second-order interference, between any two signal beams, s_j and s_k, is measured with detector D*jk*.

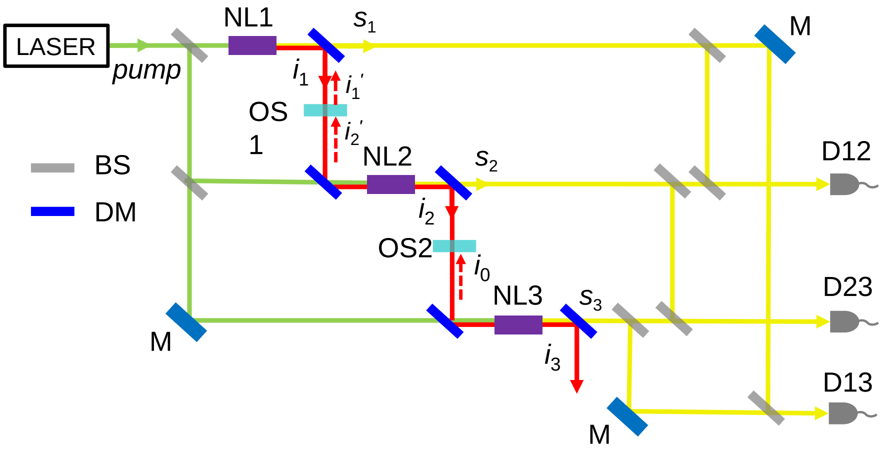

Figure S4. A schematic layout of quantum spectroscopy or imaging experiment with three SPDC crystals in a cascading arrangement. This differs from the experimental setup depicted in Figure S3 in that the planar dielectric slaps OS1 and OS2 form an optical resonator, where the propagation directions of all the idler modes as well as the two vacuum modes are orthogonal to the surfaces of OS1 and OS2. The schematic layout shown in this figure is what we consider in the main text.

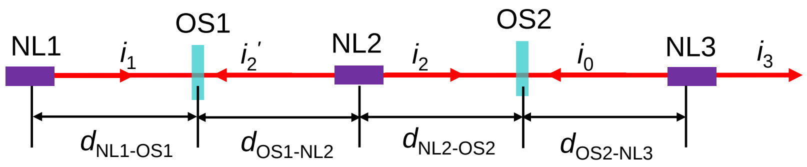

Figure S5. The relative distances between optical components. They mainly determine the phase factors gained by idler beams (see the text in this Supplementary Information).

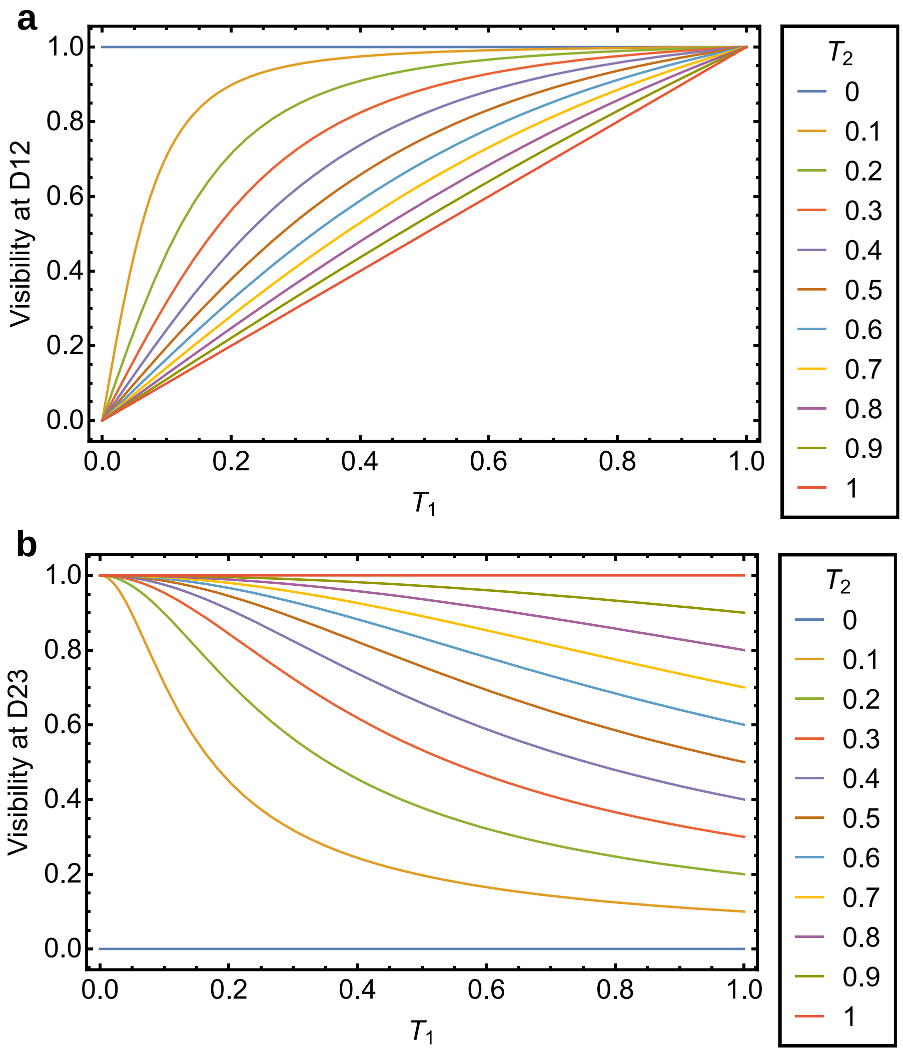

Figure S6. a Visibility of interference fringe at D12, V_{12}, with respect to amplitude transmissivity of OS1 at various T_2 from 0 to 1. **b** Visibility of interference fringe at D23, V_{23}, with respect to amplitude transmissivity of OS1 at various T_2 from 0 to 1. We assume that the three NL crystals are pumped identically.

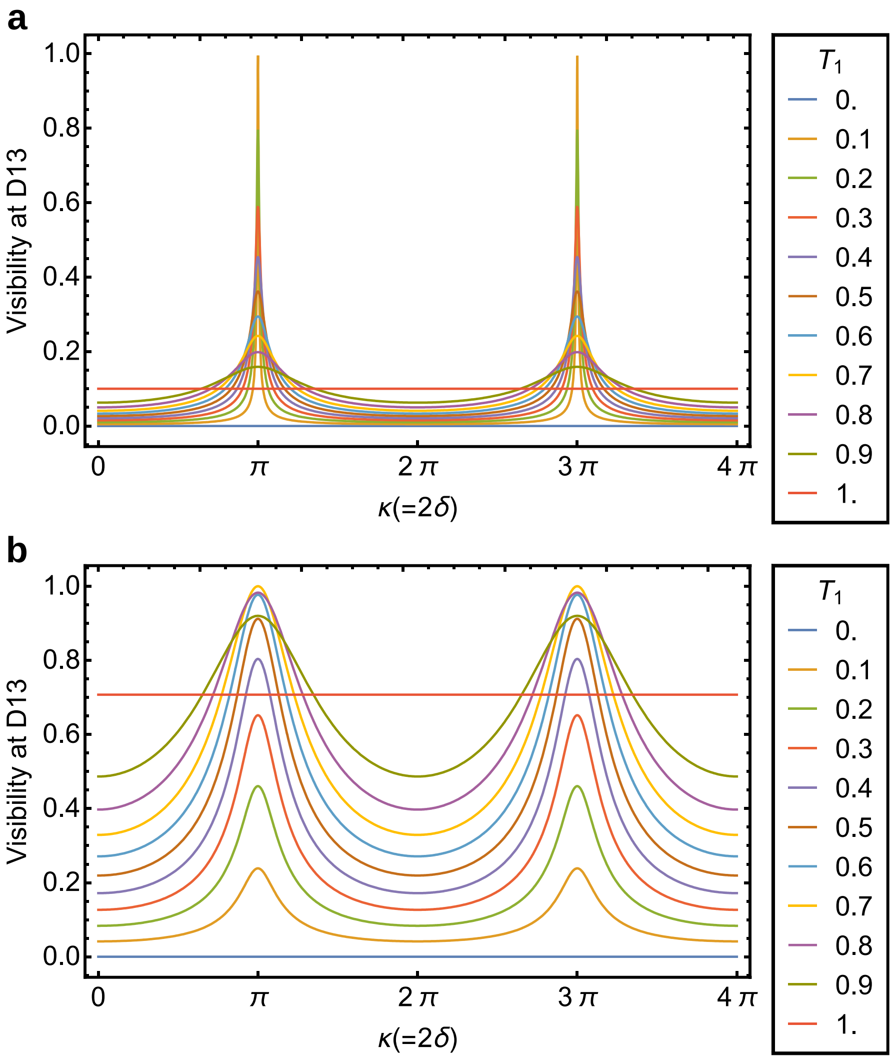

Figure S7. Visibility of interference fringe at D13, V_{13}, with respect to the phase gained by a round trip of idler beam in the cavity with $\kappa = 2\delta$, for various transmissivity(T_1) of OS1 for (**a**) $T_2 = 0.1$ and (**b**) $T_2 = 1/\sqrt{2}$. We note that the transmission becomes 1 on resonance for $T_2 = T_1$ according to the cavity input-output relation. We assume that the three NL crystals are pumped identically.
